# A DM1-doped porous gold nanoshell system for NIR accelerated redox-responsive release and triple modal imaging guided photothermal synergistic chemotherapy

**DOI:** 10.1186/s12951-021-00824-5

**Published:** 2021-03-19

**Authors:** Pengcheng Xu, Ru Wang, Wenqian Yang, Yanyan Liu, Dongsheng He, Zixuan Ye, Daquan Chen, Yuan Ding, Jiasheng Tu, Yan Shen

**Affiliations:** 1 Department of Pharmaceutics, China Pharmaceutical University, Nanjing, China; 2 Chia-Tai Tianqing Pharmaceutical Group Co. Ltd., Nanjing, China; 3 School of Pharmacy, Yantai University, Yantai, 264005 China

**Keywords:** Porous gold nanoshell, Maytansine, Chemo-thermal therapy, Multimodal imaging

## Abstract

**Background:**

Although many treatments for breast cancer are available, poor tumour targeting limits the effectiveness of most approaches. Consequently, it is difficult to achieve satisfactory results with monotherapies. The lack of accurate diagnostic and monitoring methods also limit the benefits of cancer treatment. The aim of this study was to design a nanocarrier comprising porous gold nanoshells (PGNSs) co-decorated with methoxy polyethylene glycol (mPEG) and trastuzumab (Herceptin®, HER), a therapeutic monoclonal antibody that binds specifically to human epidermal receptor-2 (HER2)-overexpressing breast cancer cells. Furthermore, a derivative of the microtubule-targeting drug maytansine (DM1) was incorporated in the PGNSs.

**Methods:**

Prepared PGNSs were coated with mPEG, DM1 and HER via electrostatic interactions and Au–S bonds to yield DM1-mPEG/HER-PGNSs. SK-BR-3 (high HER2 expression) and MCF-7 (low HER2) breast cancer cells were treated with DM1-mPEG/HER-PGNSs, and cytotoxicity was evaluated in terms of cell viability and apoptosis. The selective uptake of the coated PGNSs by cancer cells and subsequent intracellular accumulation were studied in vitro and in vivo using inductively coupled plasma mass spectrometry and fluorescence imaging. The multimodal imaging feasibility and synergistic chemo-photothermal therapeutic efficacy of the DM1-mPEG/HER-PGNSs were investigated in breast cancer tumour-bearing mice. The molecular mechanisms associated with the anti-tumour therapeutic use of the nanoparticles were also elucidated.

**Result:**

The prepared DM1-mPEG/HER-PGNSs had a size of 78.6 nm and displayed excellent colloidal stability, photothermal conversion ability and redox-sensitive drug release. These DM1-mPEG/HER-PGNSs were taken up selectively by cancer cells in vitro and accumulated at tumour sites in vivo. Moreover, the DM1-mPEG/HER-PGNSs enhanced the performance of multimodal computed tomography (CT), photoacoustic (PA) and photothermal (PT) imaging and enabled chemo-thermal combination therapy. The therapeutic mechanism involved the induction of tumour cell apoptosis via the activation of tubulin, caspase-3 and the heat shock protein 70 pathway. M2 macrophage suppression and anti-metastatic functions were also observed.

**Conclusion:**

The prepared DM1-mPEG/HER-PGNSs enabled nanodart-like tumour targeting, visibility by CT, PA and PT imaging in vivo and powerful tumour inhibition mediated by chemo-thermal combination therapy in vivo. In summary, these unique gold nanocarriers appear to have good potential as theranostic nanoagents that can serve both as a probe for enhanced multimodal imaging and as a novel targeted anti-tumour drug delivery system to achieve precision nanomedicine for cancers.

**Supplementary Information:**

The online version contains supplementary material available at 10.1186/s12951-021-00824-5.

## Background

The American Cancer Society considers breast cancer to be a major public health problem and studies have estimated that in women, the incidence of breast cancer is increasingly most rapidly among all cancers, leading to this malignancy being the second leading cause of death [1]. To date, many treatments for breast cancer have been identified, including surgical resection [2], adjunctive therapy (including chemotherapy [3], radiotherapy [4], endocrine therapy [5]), novel treatment strategies such as molecular targeted therapy [6], and hyperthermia [7]. However, it has been difficult to achieve satisfactory results with monotherapies. Hence, a combination of different tumour therapies may yield a better therapeutic effect and minimize damage to healthy tissues. However, many patients experience tumour recurrence and/or metastasis after initial treatment [8–11]. The survival rate and quality of life for breast cancer patients remains a problem for which a solution is urgently needed.

Chemotherapy is a major adjunctive breast cancer treatment option. Many drugs with various sites of action have been identified. For example, the derivative of maytansine (DM1) is a microtubule-targeted drug that binds to microtubulin and can subsequently depolymerize microtubules and arrest cells in mitosis. These powerful anti-mitotic effects are strongly toxic to many malignancies, including lung carcinoma, melanoma and breast cancer [12, 13]. Research has shown that although DM1 can effectively inhibit microtubulin aggregation and induce cell death, a narrow therapeutic window and systemic toxicity (e.g., neurotoxicity and gastrointestinal toxicity) of this drug have restricted its clinical application [14]. The identification of proper drug delivery nanocarriers might overcome these limitations since they can target the drug, thus, necessitating less of the drug to be delivered in the first place.

Nanotechnology has led to the development of nanomaterials with unique properties that can improve drug delivery, gene therapy and tumour imaging [15, 16]. Compared with single drug administration, the use of nanomaterials as carriers can protect drug from degradation and enable a sustained drug release with better therapeutic effects and fewer adverse effects [17, 18].

Furthermore, precision cancer medicine is limited not only by the achievement of an accurate diagnosis, but also by the ability to monitor tumour lesions. Therefore, theranostics, which is defined as the integration of diagnosis and treatment, has obvious advantages over single diagnostic and treatment methods. Precision medicine relies on the integration of nanomaterials into therapeutic and diagnostic approaches [19], as these materials enable monitoring of the treatment process and can provide timely feedback about the effects of treatment on a tumour. However, the diagnostic capacity of each single imaging technique is limited in terms of the penetration depth, resolution and sensitivity, and individual methods are unable to provide accurate information [20]. Therefore, current trends involve the use of a combination of multiple imaging methods with different advantages to optimize imaging effects.

Computed tomography (CT), a widely used clinical imaging modality, has a high spatial resolution and deep penetration range but characteristically low sensitivity. Photoacoustic (PA) and photothermal (PT) imaging are novel noninvasive technologies that have recently received significant attention in the context of preclinical imaging research [21–24]. PA imaging combines the deep penetration of acoustic imaging with the high sensitivity of optical imaging [25]. Meanwhile, PT imaging is a real-time technology through which radiant energy is detected and used to form pseudo-color images of the temperature distribution in the body during photothermal therapy. In this work, we designed a contrast agent consisting of a desirable vector that would integrate high resolution, deep penetration, high sensitivity and real-time diagnostic capability of these three imaging modalities.

Along these lines, gold nanoparticles provide several advantages as nanocarriers [26–31], including morphological diversity, convenient fabrication, a controllable size and easy surface modification. Gold nanoparticles are also highly biocompatible, with only minor off-site adverse effects, and exhibit unique optical properties that can be used for hyperthermia. Gold nanoparticles feature a strong decaying ability and high absorption coefficient and have been considered as a potential contrast agent in enhanced tumour diagnostic imaging approaches based on CT, PA and PT. Studies have reported that gold nanoparticles provide good contrast effects with fewer adverse effects than those associated with traditional contrast agents [26]. When designing drug delivery systems, the size and porosity of nanoparticles can influence drug loading capacity, photothermal efficiency, and biological distribution [32]. Gold nanoparticles have the function of localized surface plasmon resonance (LSPR) and can produce photothermal therapy (PTT) effect to destroy cells [33]. The LSPR spectrum of gold nanostructures depends on its geometry. Porous gold nanoshell (PGNS) have higher specific surface area, more active centers and higher drug loading capacity than solid or hollow gold nanoparticles which may suffers from the problem of drug leakage during its biological circulation [34]. In the presence of some small nanostructures, such as sharp edges, narrow depressions and pores, LSPR functions can also be enhanced to enhance photothermal efficiency [35, 36]. Thus, the porous gold nanoshell structure can improve the drug loading efficiency and photothermal efficiency compared to other reported nanostructures such as nanorods [37], nanostars [38], nanocages [39], etc.[40].

However, plain PGNSs, which lack an active targeting effect, can be easily captured by Kupffer cells and accumulate in the liver instead of the targeting area [41], which would hinder further therapeutic applications. Therefore, multifunctional PGNSs with active targeting effects and microenvironment-sensitive properties are urgently needed.

**Fig. 1:**
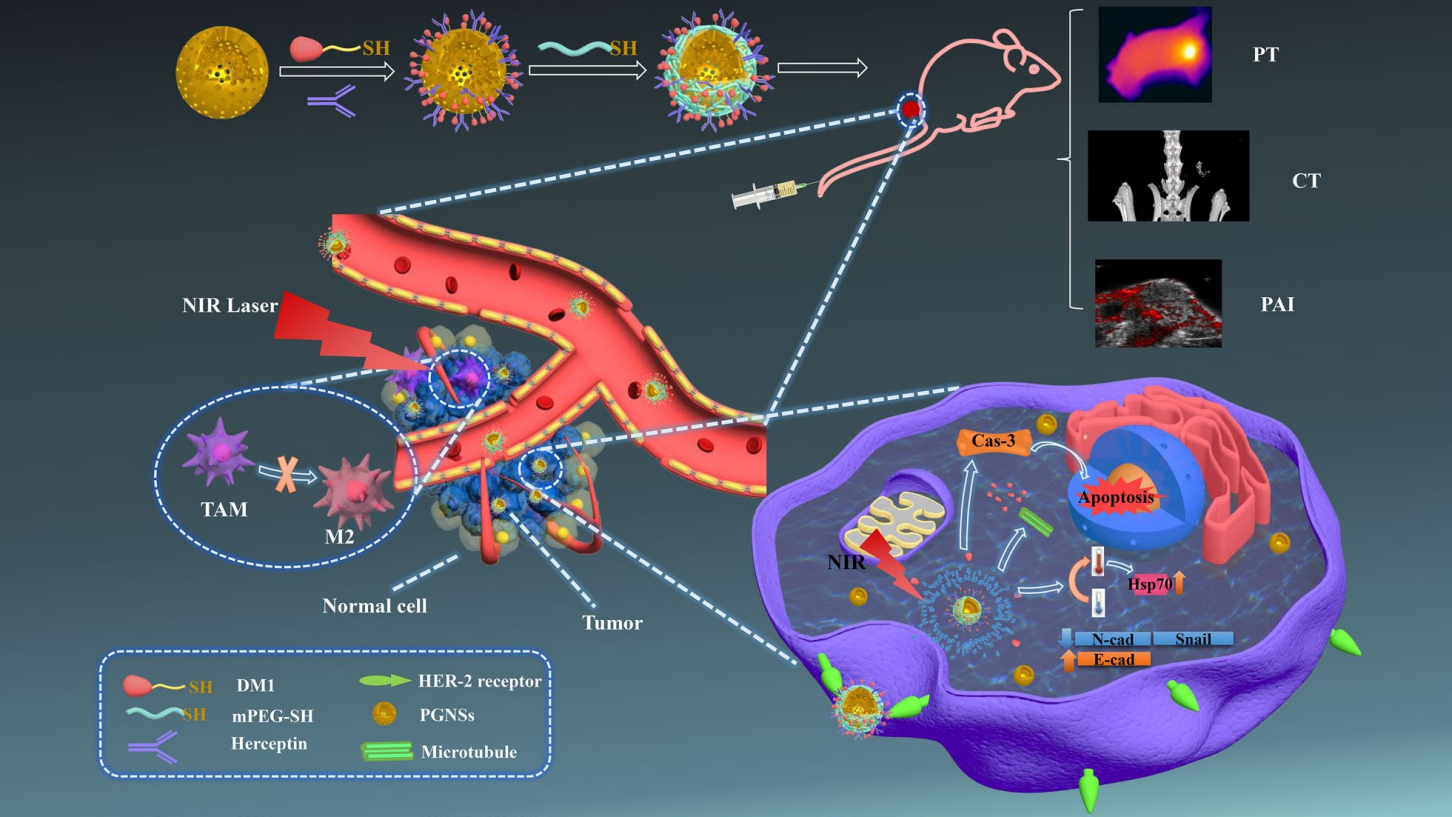
Illustration of the preparation of DM1-mPEG /HER-PGNSs and its antitumour mechanism

In consideration of these issues, here, we designed a multifunctional system based on a PGNS with tumour-targeted function that would be useful for both multimodal CT/PA/PT and chemo-thermal synergistic therapy (Fig. 1). Specifically, mPEG and trastuzumab (Herceptin®, HER), a humanized antibody that targets Her-2 and is used in combination with chemotherapy to treat patients with Her-2-overexpressing breast cancers, were attached to the surfaces of PGNSs via electrostatic interactions [42] and Au–S bonds [43]. These co-decorated nanocarriers containing the model drug DM1 (DM1-mPEG/HER-PGNSs) were proven here to serve as an excellent theranostic nanoplatform that enabled integrated, tumour-targeted, enhanced multimodal guided imaging and chemo-thermal synergistic therapy. We have additionally elucidated the mechanism of action of this chemo-thermal synergistic therapy and the molecular mechanism (including apoptotic mechanism) and anti-metastasis mechanism by which the nanoparticles function in anti-tumour applications.

## Results and discussion

### Characterization of mPEG-PGNSs, mPEG/HER-PGNSs, DM1-PGNSs and DM1- mPEG/HER-PGNSs

**Fig. 2:**
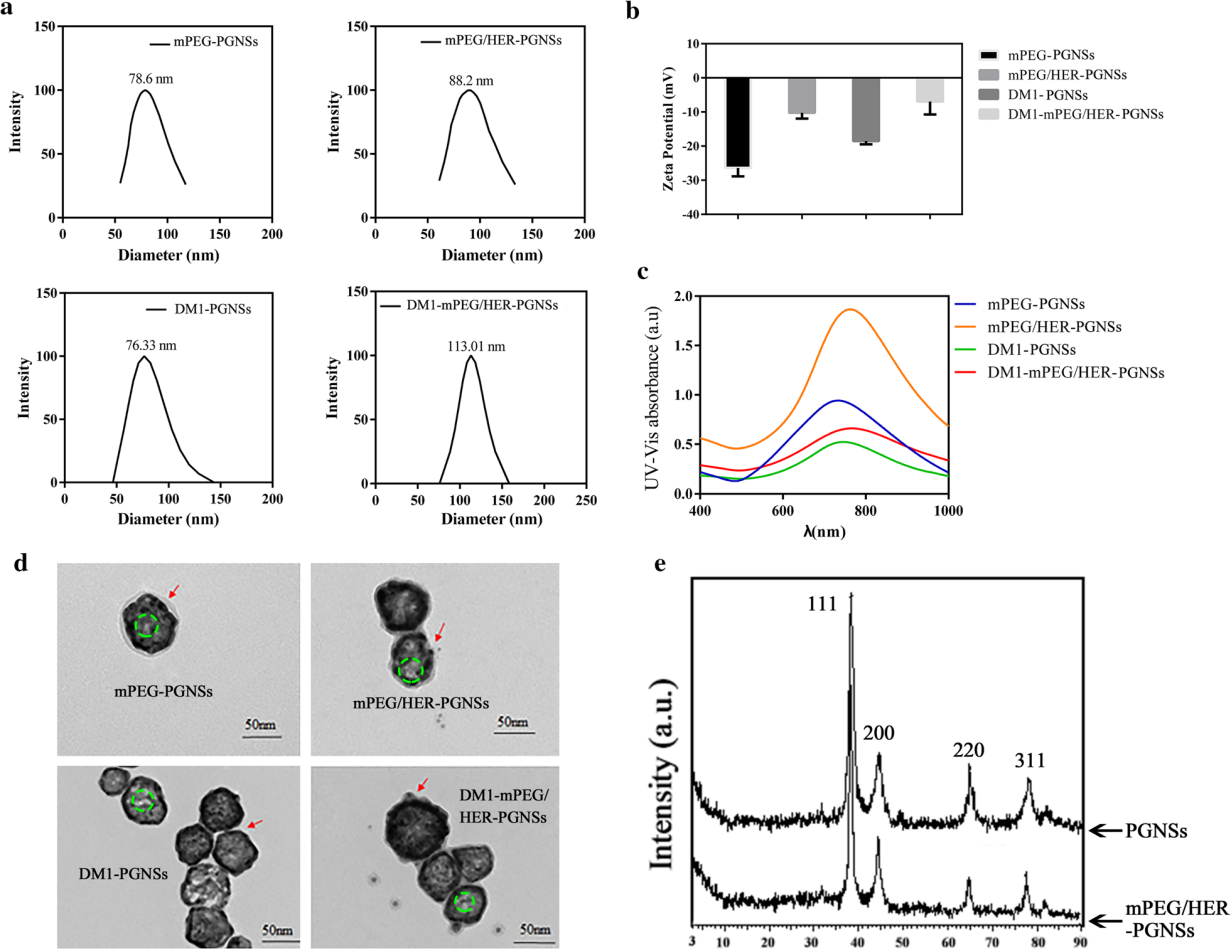
Size distribution (**a**), zeta potential (**b**), the absorption spectra (**c**) and TEM images (**d**) of mPEG-PGNSs, mPEG/HER-PGNSs, DM1-PGNSs and DM1-mPEG/HER-PGNSs (red arrows indicate the modification of mPEG and HER, respectively; green cycles indicate the porous structures); and **e** XRD patterns of PGNSs and mPEG/HER-PGNSs

The average size and polydispersity indexes (PDIs) of the mPEG-PGNSs, mPEG/HER-PGNSs, DM1-PGNSs and DM1-mPEG/HER-PGNSs (Fig. 2a) were 78.6, 88.2, 76.33 and 113.01 nm, respectively, and 0.133, 0.154, 0.150 and 0.207, respectively. The corresponding surface charges (Fig. 2b) were − 26.40 ± 2.44, − 10.21 ± 1.73, − 18.51 ± 0.97 and − 6.93 ± 3.79 mV, respectively (Fig. 2c). Compared with the unmodified PGNSs [44], the modified PGNSs featured increased sizes and zeta potentials. In addition, significant red shifts were observed in the surface plasmon resonance (SPR) absorption peaks of the modified PGNSs (Fig. 2c) relative to the unmodified PGNSs. These red shifts corresponded to disturbances in the local refractive indexes around the PGNSs due to modifications such as DM1, mPEG or HER [45].

Transmission electron microscopy (TEM) images (Fig. 2d) revealed that the modified PGNSs retained a spherical morphology with cavities inside the structure. Porous structures of gold nanoshell were obvious observed. A translucent film appeared after the nanoparticles were modified with mPEG and DM1. Moreover, modification with HER led to the appearance of a gray outer layer around the PGNSs, whereas the free HER was visible as black dots after phosphotungstic acid (1%) staining. The observed red shift and the haze-like ring in the TEM images were attributable to an increase in the particle size or zeta potential and indicated the successful modification of the PGNSs with mPEG, DM1 and HER. The connection ratio between HER and Au in the mPEG/HER-PGNSs and DM1-mPEG/HER-PGNSs were determined using a BCA standard curve (Additional file 1: Figure S2), which yielded respective values of 38.6 ± 6.8% and 35.1 ± 0.3%. In other words, HER had bound successfully to the surfaces of the PGNSs (Additional file 1: Table S2). Fourier transform infrared (FTIR) spectroscopy (Additional file 1: Figure S1) was used to define the formation of Au–S bonds between mPEG and PGNSs and revealed the existence of a weak band near 2550 cm^−1^ in mPEG, which confirmed the presence of the SH group. This band was not observed in the spectra of mPEG-PGNSs, thus, confirming the S–Au interactions [46]. X-ray diffraction (XRD) analysis was then used to confirm the crystalline structures of the synthesized PGNSs and mPEG/HER-PGNSs. The XRD pattern for the face-centered cubic structure of the PGNSs (JCPDS file no: 04–0784; Fig. 2e) depicted the characteristic Bragg’s reflection peaks at 38.21°, 44.45°, 64.86° and 77.55°, which correspond to the (111), (200), (220) and (311) planes, respectively [47]. Similar diffraction peaks were observed in the XRD pattern for the mPEG/HER-PGNSs, indicating that the dual modification did not affect the structures of the PGNSs. Recent in vivo research [48] on the mechanisms of macrophage clearance suggested that insoluble substances coated in surfactant proteins would be taken up by macrophages in a process dependent on antigen recognition.

### Photothermal conversion ability, NIR accelerated redox sensitivity-dependent release and in vitro cytotoxicity of DM1-mPEG/HER-PGNSs

**Fig. 3:**
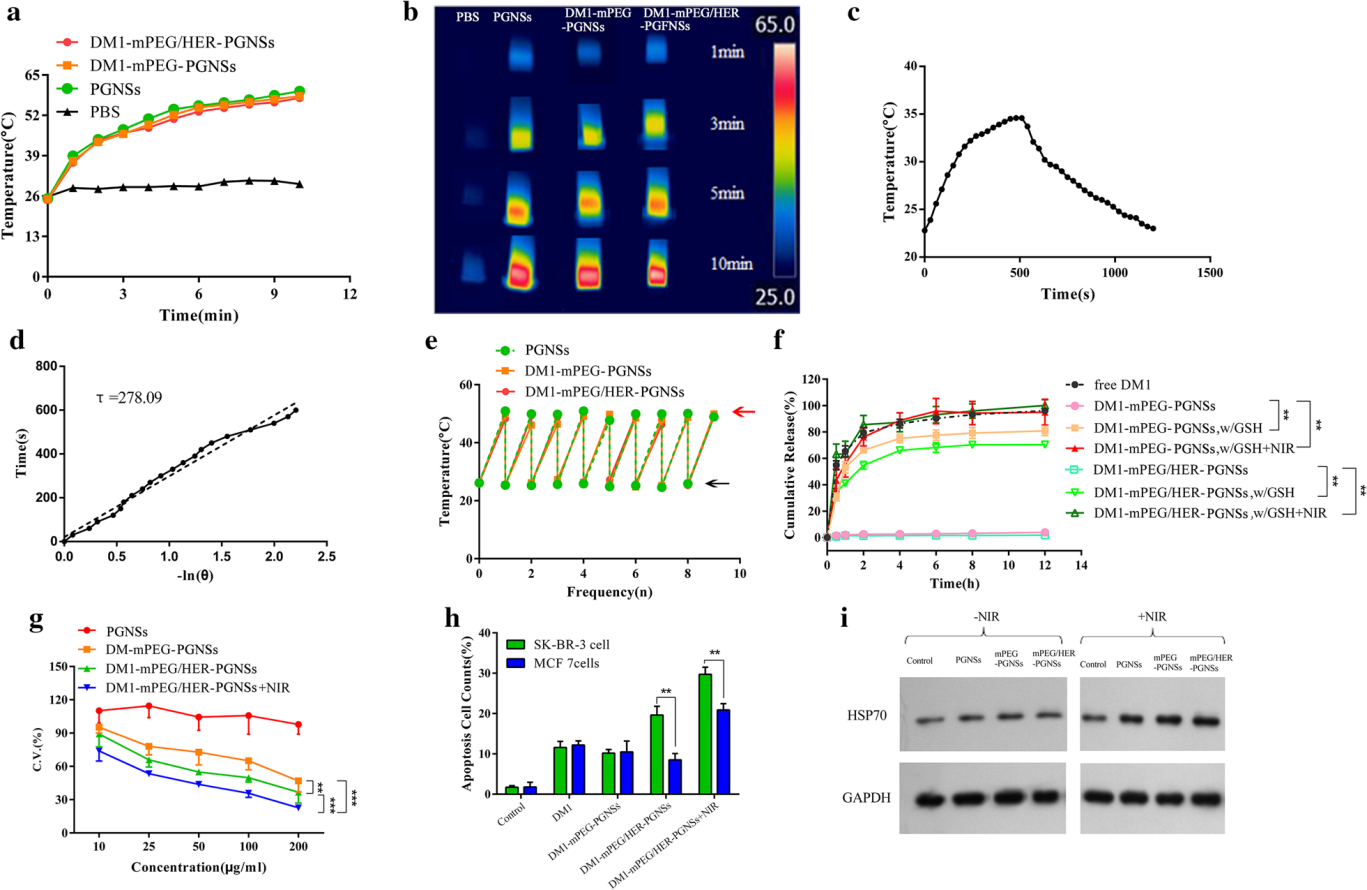
**a** Temperature changes and **b** photothermal images of PGNSs, DM1-mPEG-PGNSs and DM1-mPEG/HER-PGNSs after NIR irradiation for 10 min; **c** Temperature change of PGNS in aqueous solution with a 808 nm laser (3 W cm^−2^), the irradiation lasted for 500 s then was shut off; **d** Plot of the cooling time versus –ln(θ) obtained from the cooling stage in **c**; **e** temperature increments of PGNSs, DM1-mPEG-PGNSs and DM1-mPEG/HER-PGNSs after nine repeated irradiations (black arrows indicate temperature before illumination and red arrow indicates temperature after illumination); **f** in vitro release of DM1 of free DM1, DM1-mPEG-PGNSs and DM1-mPEG/HER-PGNSs in phosphate-buffered saline 7.4 at 37 °C; **g** SK-BR-3 cell viability when exposed to PGNSs, DM1-mPEG-PGNSs, DM1-mPEG/HER-PGNSs and DM1-mPEG/HER-PGNSs + NIR after 24 h; **h** Different apoptosis cell counts after treating with different DM1 formulations; and **i** Western blot analysis of HSP70 in SK-BR-3 cells. (^**^p < 0.01, ^***^p < 0.001)

The temperatures (Fig. 3a) of DM1-mPEG-PGNSs and DM1-mPEG/HER-PGNSs (Au: 15 μg mL^−1^) increased by approximately 32 °C after the respective modification and tri-modification when compared with that of the unmodified PGNSs, and these changes were not accompanied by a decrease in the photothermal conversion efficiency. Similar results (Fig. 3b) were obtained when using an infrared thermal image instrument, which demonstrated that the DM1 modification would not affect the photothermal conversion abilities of the PGNSs. Hence, the photothermal conversion abilities of both the modified and unmodified PGNSs could facilitate hyperthermia relatively rapidly, thus, enabling a thermotherapeutic effect and the global or local ablation of tumour tissues at temperatures of 39–42 °C [49].

The approximate photothermal conversion efficiency (η) of PGNSs exposed to an 808-nm laser was 22.43%. Notably, this value was higher than that of gold nanoparticles alone (11%) [50] and similar to the values of some known photothermal agents such as gold nanorods (21%) [51], which are highly toxic due to the use of surfactants such as cetyltrimethylammonium bromide (CTAB) and Cu_9_S_5_ nanocrystals (~ 25.7%) [52] and are synthesized at high pressures and temperatures.

To investigate the thermal stability of PGNSs before and after modification, intermittent NIR illumination was used to determine whether the photothermal conversion would decline relative to its initial state. As shown in Fig. 3e, the temperatures of PGNSs, DM1-mPEG-PGNSs and DM1-mPEG/HER-PGNSs increased by 26 °C during the first cycle and returned to the same level in subsequent cycles without attenuation. These results prove that both modified and unmodified PGNSs could achieve a hyperthermic effect even after several rounds of NIR illumination.

Reports have described greatly increased concentrations of molecules such as glutathione (GSH), ascorbic acid and cysteine in tumour tissues [53], and consequently, tumour cells have a higher redox status (20 mM). In this study, GSH was used to simulate different degrees of the reduction status in vivo, while NIR illumination was used to investigate the effect of GSH on the release of DM1 due to the photothermal conversion effect. As shown in Fig. 3f, significantly more DM1 was released from DM1-mPEG-PGNSs and DM1-mPEG/HER-PGNSs in the presence of GSH than in the absence of GSH (^**^p < 0.01). Possibly, DM1 was linked to PGNSs via a Au–S chemical bond, which was very stable and did not degrade to release DM1. Once GSH was added to the release medium, however, it functioned as a reducing agent to enable the exchange of therapeutic ligands from the surfaces of the PGNSs and yielded a thiol group exchange reaction and GSH-induced drug release [54, 55]. Approximately 80% and 70% of the bound DM1 was released from the DM1-mPEG-PGNSs and DM1-mPEG/HER-PGNSs, respectively, at 12 h after the addition of GSH.

Furthermore, we explored the profiles of DM1 released from DM1-mPEG-PGNSs and DM1-mPEG/HER-PGNSs after GSH and NIR illumination (Fig. 3f). The cumulative release of DM1 was almost complete (95% and 98% for DM1-mPEG-PGNSs and DM1-mPEG/HER-PGNSs, respectively) after NIR irradiation for 5 min, indicating that this intervention could trigger the rapid release of DM1. In summary, the above results strongly suggest that both the photothermal conversion ability of PGNSs and the Au–S bonds are sensitive to the reduction status of the tumour environment, and this sensitivity promotes the release of DM1. The selective release of drugs at the tumour target site is important for drug efficacy and safety in both passive and active targeting approaches. Here, the fabricated redox-sensitive DM1-loaded formulations responded specifically to the tumour microenvironment, remained stable in the blood circulation and enabled the specific release of drugs at the tumour site. These nanoparticles could therefore enhance tumour targeting and promote cellular uptake while reducing drug leakage during delivery [56].

To further investigate the antitumour activity associated with the chemo-thermal therapeutic synergistic treatment and the targeting effect of the HER modification, four groups of mPEG/HER-PGNSs were tested against SK-BR-3 and MCF-7 cells, which respectively express high and low levels of the Her-2 receptor (verified in Additional file 1: Table S3 and Figure S3). The treated cells were then subjected to an MTT assay. As shown in Fig. 3g, unmodified PGNSs exhibited little cytotoxicity, and the SK-BR-3 cell viability rates were as high as 90% even at an Au concentration of 200 μg mL^−1^. Meanwhile, the viability of LO2 human hepatocyte cells also exceeded 90% at Au concentrations below 50 μg mL^−1^ (Additional file 1: Figure S5A). This indicated that the modification with mPEG or HER did not create additional toxicity of the PGNSs. These modified PGNSs induced higher levels of cytotoxicity in SK-BR-3 cells (lowest survival viability: 36%) relative to MCF-7 cells (lowest survival viability: 57%; ^*^p < 0.05; Additional file 1: Figure S5B). Moreover, the combination of the DM1-mPEG/HER-PGNSs and NIR illumination achieved a comprehensive chemo-thermotherapeutic effect in both types of breast cancer cells. Additionally, the IC_50_ value of the synergistic index for the DM1-mediated chemotherapy and PGNS-mediated photothermal therapy in SK-BR-3 cells was 0.45164 (Additional file 1: Figure S5B-D), indicating a synergistic effect. MCF-7 cells were used to verify the higher inhibition effect of the Herceptin modified drug-loaded PGNSs (Additional file 1: Figure S5E). Dual modification with mPEG and HER led to an increase in the ingestion of DM1-mPEG/HER-PGNSs by the SK-BR-3 cells, which strongly expressed Her-2. Moreover, the combined treatment with DM1-mPEG/HER-PGNSs and NIR yielded total cell apoptosis rates that were approximately 8.4-fold and 12.5-fold higher than those in the control group for both MCF-7 and SK-BR-3 cells, respectively (Fig. 3h and Additional file 1: Figure S5F&G). This apparent synergistic effect in both breast cancer cell lines corresponded to the results from the cytotoxic study. As shown in Fig. 3i, the expression of the 70-kDa heat shock protein (HSP70) was examined via western blotting, and higher levels were observed in the cells exposed to NIR with unmodified PGNSs and modified PGNSs (mPEG-PGNSs and mPEG/HER-PGNSs), whereas extremely low levels of HSP70 expression were observed in the remaining groups. These results confirm that the temperature increase mediated by both the unmodified and modified PGNSs was sufficient to induce stress and transient thermotolerance in the cells [57].

### Stability study

**Fig. 4:**
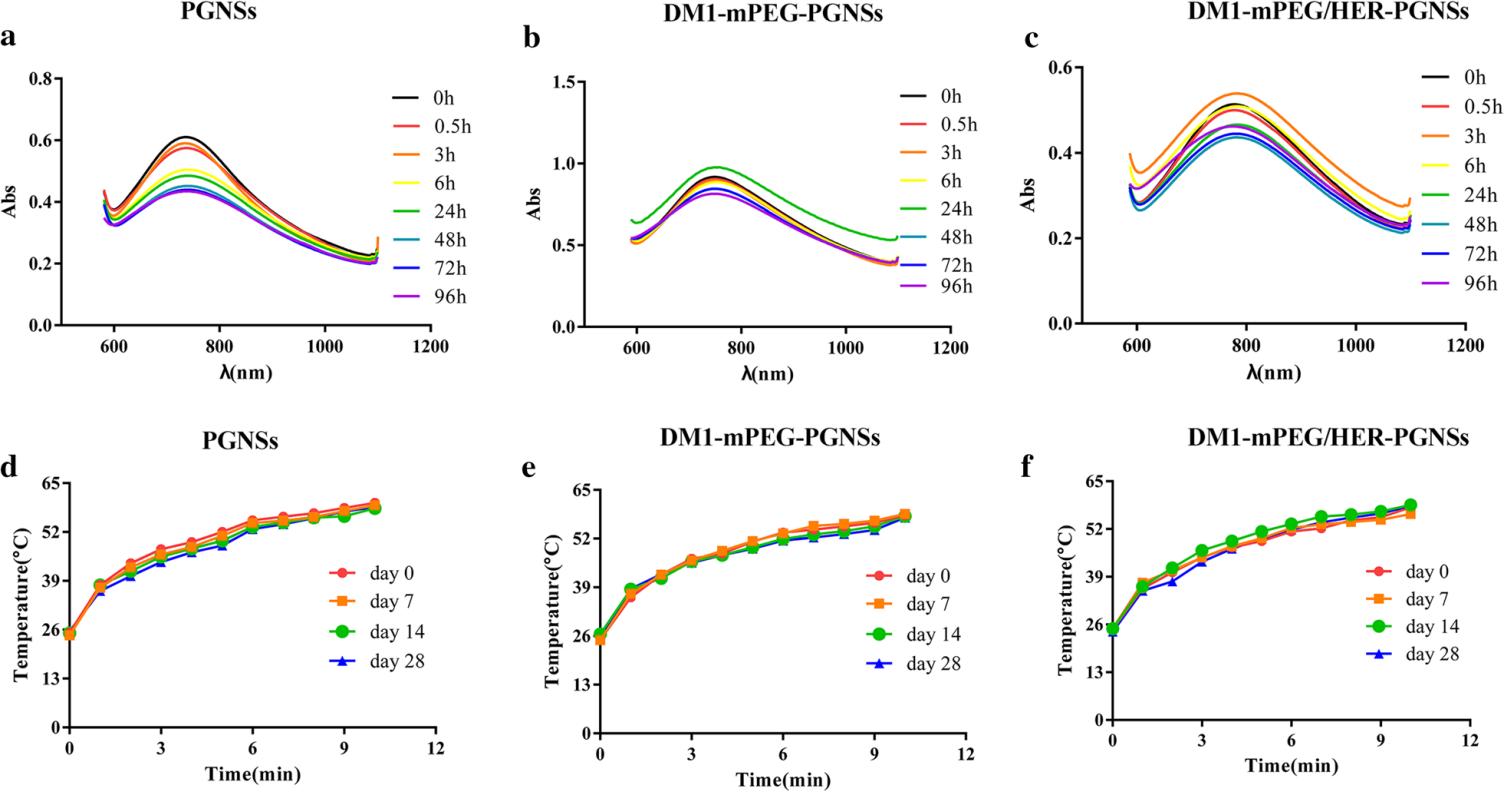
The absorption spectra of PGNSs (**a**), DM1-mPEG-PGNSs (**b**) and DM-mPEG/HER-PGNSs (**c**) in the presence of serum; Temperature changes of PGNSs (**d**), DM1-mPEG-PGNSs (**e**) and DM1-mPEG/HER-PGNSs (**f**) during storage at 4 °C for 28 days

Stabilisation at the nano scale is a key contributor to the uniquely high surface areas of metal nanoparticles. Hence, we investigated the stability of PGNSs before and after modification. Using FBS to mimic the serum environment in vivo, the stability was evaluated based on variations in the SPR absorption spectra. As shown in Fig. 4a–c, the uncoated PGNSs, DM1-mPEG-PGNSs and DM1-mPEG/HER-PGNSs all appeared to be stable in serum, as indicated by the lack of obvious changes in SPR absorption. The mPEG-PGNSs and mPEG/HER-PGNSs were also stable in serum (Additional file 1: Figure S4). The PGNSs, DM1-mPEG-PGNSs and DM1-mPEG/HER-PGNSs likely possessed a negative charge that would enhance stability by repelling negative biomacromolecules in the serum. The above results prove that the structural integrity of the nanoparticles could be maintained.

Previously, we proved that PGNSs could remain stable after several rounds of NIR illumination, without obvious changes in size, SPR absorption or photothermal conversion ability [44]. Here, we investigated the stability of the photothermal conversion ability of DM1-loaded PGNSs during long-term storage at 4 °C. As shown in Fig. 4d–f, during storage for 28 days at 4 °C, no significant declines were observed in the photothermal conversion abilities of the PGNSs, DM1-mPEG-PGNSs or DM1-mPEG/HER-PGNSs (p > 0.05). To investigate whether the increased temperature induced by NIR irradiation during photothermal therapy would affect the stability of released DM1, we also measured the changes in the DM concentration before (~ 20 μg mL^−1^) and after incubation for 5 min in a 46 °C water bath. The DM1 concentration before heating was 20.02 ± 0.006 μg mL^−1^, and increased to 19.98 ± 0.008 μg mL^−1^ after heating, suggesting that the NIR irradiation-induced increase in temperature would not affect the stability of the drug.

### In vitro* cellular uptake kinetics*

**Fig. 5:**
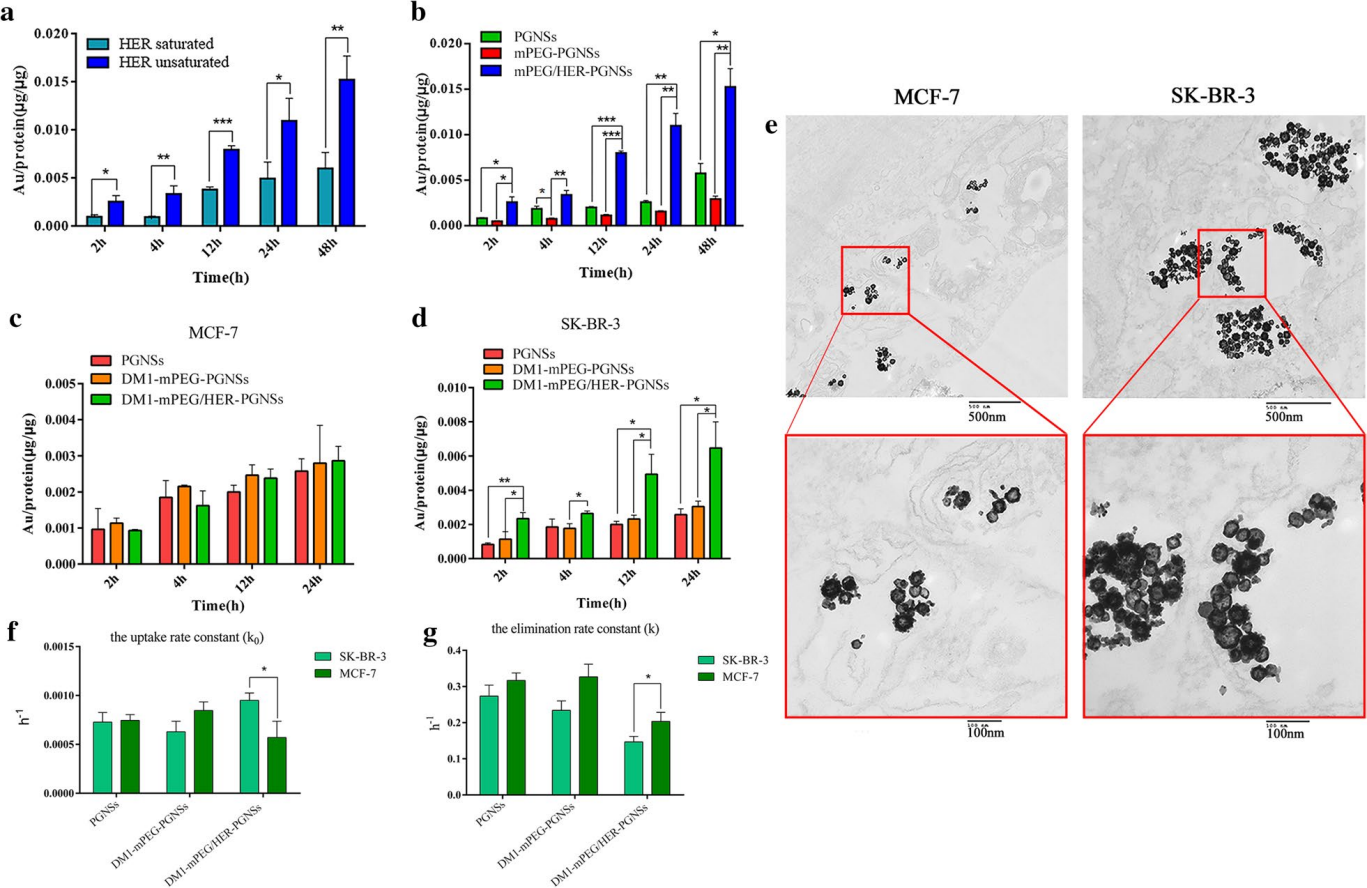
**a** Quantitative comparation of mPEG/HER-PGNSs internalized into SK-BR-3 cells with or without Her-2 antibody blocking; **b** Quantitative comparation of PGNSs, mPEG-PGNSs and mPEG/HER-PGNSs internalized into SK-BR-3 cells at 2, 4, 12, 24 h and 48 h using ICP-MS; **c** Quantitative comparation of PGNSs, DM1-mPEG-PGNSs and DM1-mPEG/HER-PGNSs internalized into MCF7 cells and **d** SK-BR-3 cells at 2, 4, 12, and 24 h using ICP-MS; and **e** TEM thin sections of cellular uptake of DM1-mPEG/HER-PGNSs in MCF7 cells and SK-BR-3 cells after 12 h (Scale bar = 500 nm, 100 nm); **f** k_0_ and **e** k values for cellular uptake and elimination in SK-BR-3 cells and MCF-7 cells. (^*^p < 0.05,^**^p < 0.01,^***^p < 0.001)

Inductively coupled plasma mass spectrometry (ICP-MS) was used to quantify the cellular uptake in vitro. First, competitive inhibition of HER2 was investigated, as shown in Fig. 5a. Pre-blocking of the cells using an excess of HER led to a large decrease in the cellular uptake of mPEG/HER-PGNSs, indicating that this therapeutic monoclonal antibody weakened the targeting effect of mPEG/HER-PGNSs by blocking HER2. This finding validates the ability of mPEG/HER-PGNSs to specifically target HER2.

The cellular uptake of non-DM1-loaded PGNSs (PGNSs, mPEG-PGNSs and mPEG/HER-PGNSs) by SK-BR-3 cells is shown in Fig. 5b. The cellular uptake of the PGNSs increased in a time-dependent manner, and increased internalisation of mPEG/HER-PGNSs relative to that of PGNSs was observed at the same incubation time, suggesting that the inclusion of HER improved the targeting efficiency. In contrast, the cellular uptake of mPEG-PGNSs decreased. The endocytosis of some inorganic nanoparticles (such as gold nanoparticles) is mainly dependent on the adsorption of serum proteins on the particle surfaces [58]. PEGylation reduced this serum protein adsorption on PGNSs, leading to a decrease in cellular uptake. The scavenger receptor, a group of cell surface proteins that actively facilitate the uptake of a variety of molecules (predominantly negatively charged macromolecules), is the main route of gold nanoparticle uptake [59]. However, Van Haute et al. [60] observed suppressed interactions between scavenger receptors and gold nanoparticles after PEGylation, which reduced cellular uptake. In the present study, the maximum uptake of PEGylated PGNSs by SK-BR-3 cells was observed when the nanoparticles were co-labelled with HER, enabling specific targeting of HER2.

The cellular uptake of DM1-loaded PGNSs is shown in Fig. 5c, d. In both SK-BR-3 and MCF-7 cells, the cellular uptake of PGNSs, DM1-mPEG-PGNSs and DM1-mPEG/HER-PGNSs exhibited a time-dependent trend. In SK-BR-3 cells, the uptake of DM1-mPEG/HER-PGNSs was greater than that of PGNSs or DM1-mPEG-PGNSs and was approximately double the uptake of DM1-mPEG/HER-PGNSs by MCF-7 cells after 24 h. To confirm the uptake and intracellular distribution of DM1-mPEG/HER-PGNSs, both types of cells were incubated with the PGNS formulations for 12 h and then observed using TEM (Fig. 5e). PGNSs were visible in the cytoplasm of both MCF-7 and SK-BR-3 cells, which confirmed successful cellular uptake. Moreover, the cellular uptake was noticeably greater in SK-BR-3 cells than in MCF-7 cells. These findings support the remarkable targeting capability of DM1-mPEG/HER-PGNSs due to the active targeting effect of HER.

Given the presence of a large number of nanoparticles in the culture medium, cellular uptake was assumed to occur at a constant rate. Simultaneously, a substance internalised into the cells was eliminated according to the first-order kinetics equation [61, 62].

Over time, the change in the intracellular content of a substance could then be calculated as the algebraic sum of the intake and elimination. Therefore, the relationship between intracellular nanoparticle accumulation and time could be determined using Eq. 1

1 $$ \frac{{{\text{d}}X}}{{{\text{dt}}}} = {\text{k}}_{0} - {\text{k}}X. $$

After the Laplace transformation, the equation was described is as follows (Eq. 2):

2 $$ X = \frac{{{\text{k}}_{0} }}{{\text{k}}}\left( {{1} - {\text{e}}^{{ - {\text{kt}}}} } \right) $$

where X (μg/μg) is the amount of the internalized nanoparticles per microgram of protein at time t, k_0_ (h^−1^) is the uptake rate constant and k (h^−1^) is the elimination rate constant.

The uptake of PGNSs, DM1-mPEG-PGNSs and DM1-mPEG/HER-PGNSs by SK-BR-3 cells and MCF-7 cells should be in accord with the calculations determined using Eq. 1. The k0 and k values were calculated after nonlinear fitting using GraphPad Prism 6 (GraphPad Software Inc., San Diego, CA, USA), and the results are shown in Fig. 5f–g. The rates of PGNS absorption and elimination by SK-BR-3 and MCF-7 cells were similar. The rates of DM1-mPEG-PGNS absorption and elimination were approximately 75% slower in SK-BR-3 cells than in MCF-7 cells. However, DM1-mPEG/HER-PGNSs were taken up by SK-BR-3 cells at a rate 1.68 times the rate of uptake by MCF-7 cells, and the rate of elimination by SK-BR-3 cells was only 75% of the rate of elimination by MCF-7 cells. In summary, dual modification of the PGNSs by mPEG and HER increased both the total uptake and speed of uptake by SK-BR-3 cells.

### In vivo fluorescence imaging and biodistribution

**Fig. 6:**
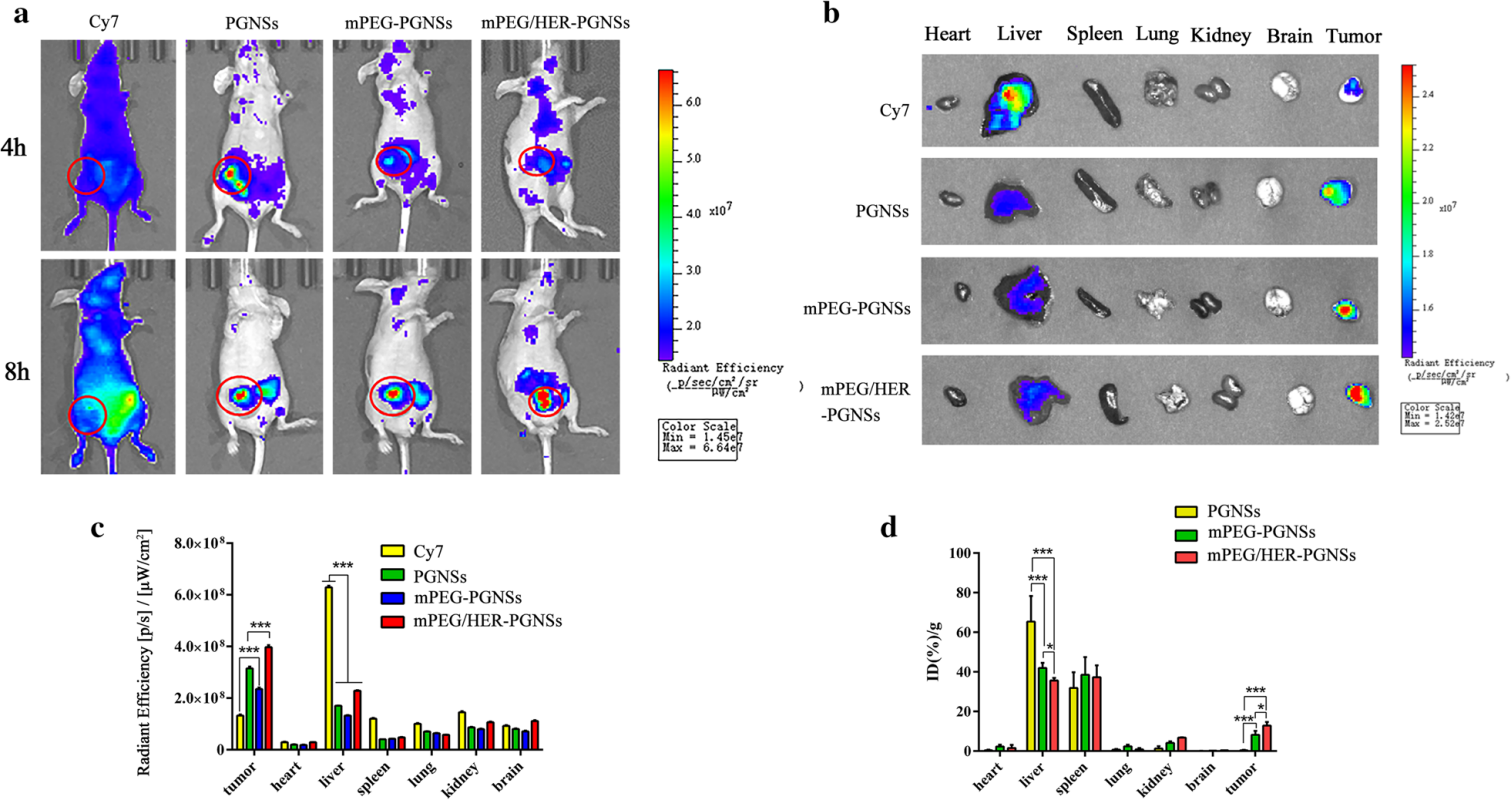
**a** In vivo imaging of SK-BR-3 bearing mice after being administered with the formulations; **b** Ex vivo imaging of the tumour and other main organs; **c** semi-quantitative fluorescence intensity of in vivo imaging (**a**); and **d** Au content in each tissue using ICP-MS. Red cycles represent tumour site(^*^p < 0.05, ^***^p < 0.001)

To evaluate the biodistribution and tumour targeting efficiencies of the different modified PGNS formulations in vivo, the Au content in different tissues were detected using in vivo fluorescence imaging and ICP-MS. Notably, mPEG/HER-PGNSs were associated with both the strongest fluorescence intensity at the tumour site, as well as the highest Au content detected by ICP-MS (Fig. 6). Interestingly, the fluorescence intensity was much lower in the liver than in the tumour (Fig. 6b), whereas the Au concentration was higher in the liver than in tumour, according to the ICP-MS results (Fig. 6d). This phenomenon was attributed to the ability of a large accumulation of gold nanoparticles to quench the fluorescence of SH-PEG-Cy7, which would reduce the fluorescence in the liver and spleen [63]. In contrast, the high level of GSH in the tumour microenvironment would release SH-PEG-Cy7 from the PGNSs, and thus the fluorescence would not be quenched. The PGNSs could be delivered to tumours via the enhanced permeability and retention (EPR) effect. After modification with mPEG, and especially co-modification with mPEG and HER, the tumour targeting efficiencies of the PGNSs increased relative to that of the unmodified PGNSs. Ex vivo imaging analyses of the tumour and other main tissues also proved the existence of an active and passive targeting-enhanced delivery system. The distribution of the three PGNS preparations in vivo was investigated using ICP-MS. As shown in Fig. 6d, compared with the unmodified PGNSs, the PGNSs coated with mPEG and mPEG/HER exhibited greatly enhanced tumour-targeting abilities, with respective relative increases of approximately 22 and 35 times. Compared with DM1-mPEG-PGNS, DM1-mPEG/HER-PGNS also exhibited a significantly improved in vivo targeting ability (*p < 0.05). Their accumulation in the liver and kidney indicated that the mPEG/HER-PGNSs were mainly metabolised by the liver. The accumulation in the liver and kidney indicated that the mPEG/HER-PGNSs were mainly metabolized by the liver. The observed simultaneous decrease in the liver uptake, an inevitable problem associated with nanocarriers, suggested that the mPEG/HER-PGNSs could significantly increase drug accumulation in the tumour.

### Multimodal CT/PA/PT imaging in vivo

**Fig. 7:**
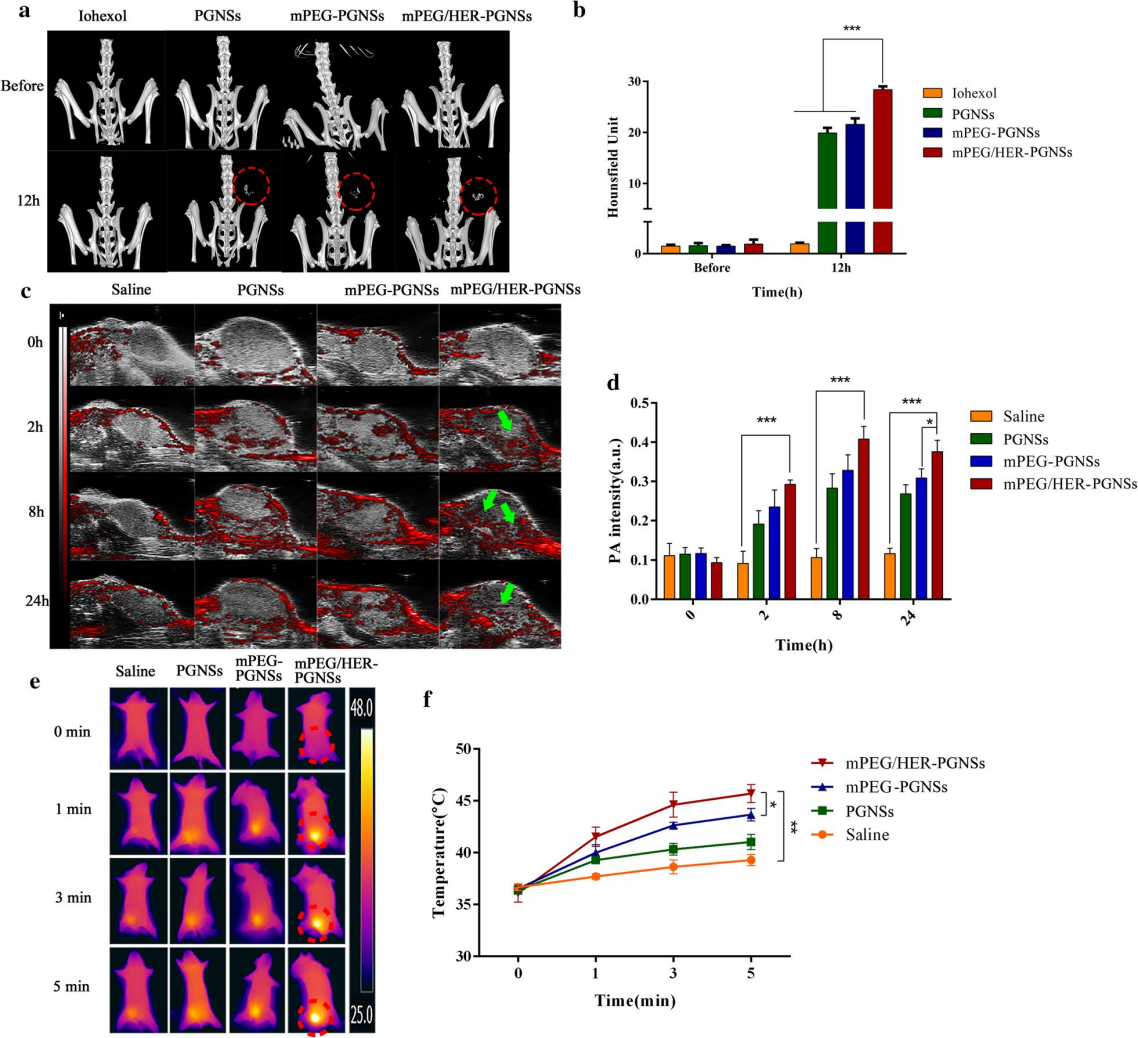
**a** In vivo CT imaging of orthotopic tumour-bearing mice before and 12 h after intravenous injection of iohexol, PGNSs, mPEG-PGNSs and mPEG/HER-PGNSs (red circles represent the tumour site); **b** CT values of the orthotopic tumour-bearing mice at different time points; **c** in vivo PA imaging of orthotopic tumour-bearing mice before and 2, 8, 24 h after intravenous injection of saline, PGNSs, mPEG-PGNSs and mPEG/HER-PGNSs (green arrows represent the PA signal); **d** PA intensity of the orthotopic tumour-bearing mice at different time points; **e** in vivo PT imaging with a NIR laser at 0, 1, 3 and 5 min of orthotopic tumour-bearing mice 24 h after intravenous injection of saline, PGNSs, mPEG-PGNSs and mPEG/HER-PGNSs (red circles represent the tumour site); and **f** temperature changes of the orthotopic tumour-bearing mice during NIR irradiation at different points. (^*^p < 0.05, ^**^p < 0.01, ^***^p < 0.001)

Due to high absorption coefficients and strong decay abilities of PGNSs, the mPEG/HER-PGNSs could be used as a novel agent for CT and PA imaging and could enable simultaneous PT imaging during photothermal treatment as a form of real-time treatment monitoring. The targeted enhanced CT/PA/PT imaging effects of mPEG/HER-PGNSs in vivo were explored in orthotopic tumour-bearing mice. A previous study revealed that PGNSs exhibited a significant contrast effect in vivo [44]. In this study, CT images were acquired using a CT scanner (Fig. 7a), and a region of interest was placed on the tumour site to obtain an average Hounsfield Units value (Fig. 7b). The tumour region exhibited obvious enhancement after injection with the mPEG/HER-PGNSs. This enhancement was maintained for up to 12 h, consistent with our previous study [44]. However, no apparent tumour site contrast was observed in the group injected with iohexol.

The PA images (Fig. 7c) revealed that the brightness in the tumour area increased significantly after PGNS administration. This contrast increased gradually over time and was maintained at 24 h after injection. Similarly, the results from PT imaging (Fig. 7e) during NIR-mediated photothermal therapy demonstrated a more rapid and obvious increase in temperature in the tumours exposed to both DM1-mPEG/HER-PGNSs and NIR treatment than those treated with saline and NIR. Accordingly, this combination enabled the achievement of a suitable temperature for tumour cell apoptosis induction without damaging the normal tissues [64, 65]. A combined analysis of the three imaging statistics (Fig. 7b, d, f) suggested that mPEG/HER-PGNSs accumulated at higher levels in the tumour than did the other modified PGNSs, due to the dual-target mechanism of EPR and active antibody targeting, and this enabled better multimodal imaging. These results clearly suggest that mPEG/HER-PGNSs could be used as a targeted CT/PA/PT contrast agent in the context of precision cancer medicine.

### In vivo antitumour efficacy

**Fig. 8:**
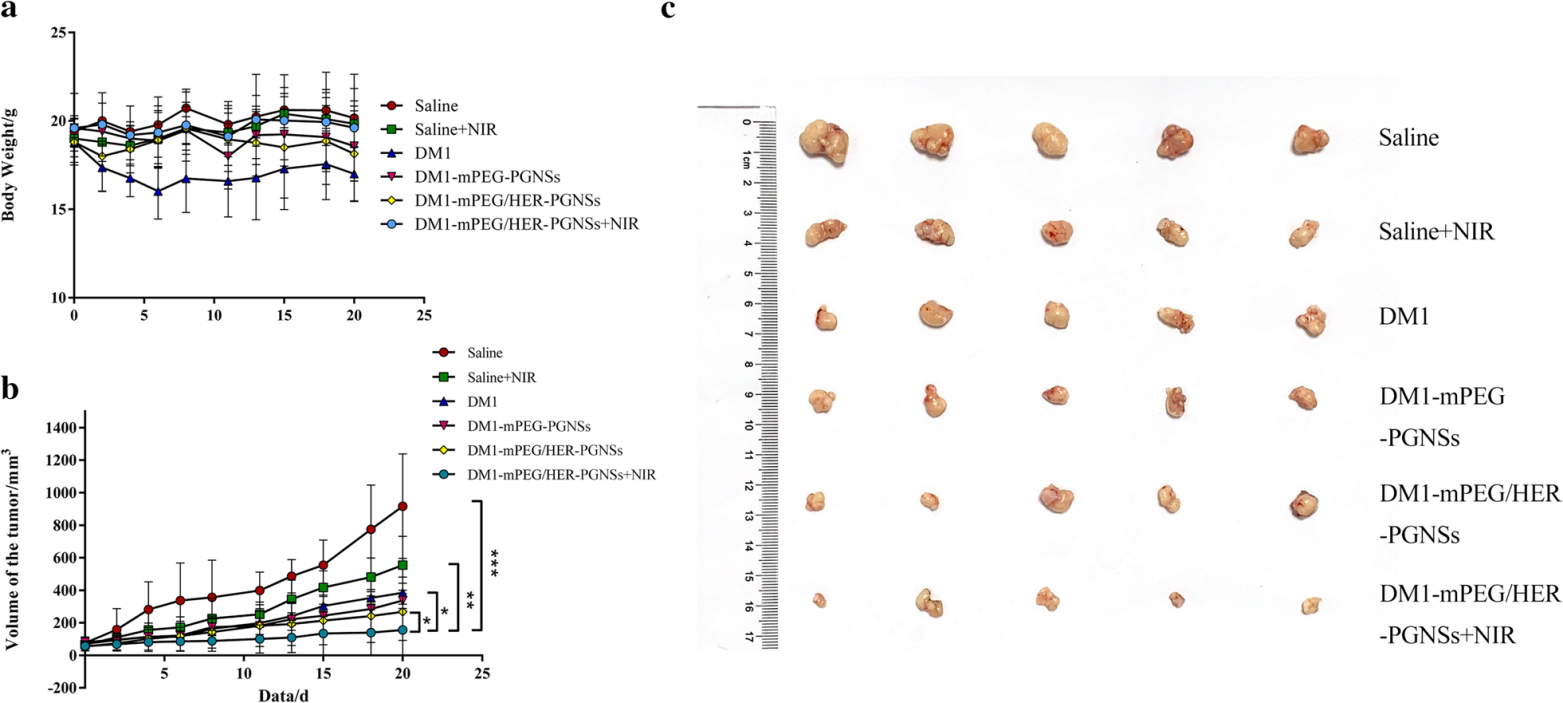
Antitumour effect to SK-BR-3 tumour bearing nude mice (n = 5). **a** Average tumour volumes with different formulations. **b** Body weight of mice with various treatments. **c** Images of the dissected tumours after a 20-day therapy in each group. (^*^p < 0.05, ^**^p < 0.01, ^***^p < 0.001)

To investigate the synergistic chemo-photothermal therapeutic efficacy of the DM1-mPEG/HER-PGNSs against breast cancer in vivo, six formulations were injected intravenously into orthotopic SK-BR-3 tumour-bearing nude mice (n = 5) in which the tumour volumes had reached approximately 60 mm^3^. Notably, only slight losses in body weight were observed in all groups (Fig. 8a) except the DM1 group, suggesting that our PGNS formulation reduced the toxicity of DM1. We also measured the tumour volumes every other day (Fig. 8b) to evaluate the in vivo anti-tumour effects of the different formulations relative to saline as the negative control. Next, the efficacy of NIR illumination was evaluated in vivo in tumour-bearing mice. Notably, mice treated with DM1-mPEG/HER-PGNSs + NIR exhibited a slower increase in tumour volume than the other treated groups. No significant difference in tumour volume was observed between the DM1-mPEG/HER-PGNS- and DM1-mPEG-PGNS-treated groups, which may have been due to the release of DM1 from the PGNSs. Although HER modification and HER2-mediated active targeting enhanced PGNS accumulation at the tumour site (Fig. 8a, b), the release of DM1 in GSH was slower than that of DM1-mPEG-PGNSs without NIR illumination due to the protein corona [66] (Fig. 3f), which compensated for the increased tumour accumulation and resulted in a non-distinctive anti-tumour effect. The speed of DM1 release by both DM1-mPEG/HER-PGNSs and DM1-mPEG-PGNSs increased in response to NIR stimulation, with no obvious intergroup difference. However, as greater intra-tumour accumulation was observed with DM1-mPEG/HER-PGNSs than with DM1-mPEG-PGNSs, the DM1 released by the former particles after NIR irradiation yielded relatively better anti-tumour effects. At 20 days post-administration, the saline + NIR, DM1, DM1-mPEG-PGNSs, DM1-mPEG/HER-PGNSs and DM1-mPEG/HER-PGNSs + NIR groups exhibited tumour growth inhibition rates of 39.47%, 58.1%, 63.24%, 70% and 83.9%, respectively, relative to the saline-treated group. These decreases in tumour growth were confirmed using photographs of the tumours (Fig. 8c), which were dissected 20 days after treatment. Meanwhile, the results of alanine aminotransferase (ALT), aspartate aminotransferase (AST), blood urea nitrogen (BUN) and creatinine (CREA) and hematoxylin and eosin staining (Additional file 1: Figure S7) suggested that this delivery system did not cause marked damage and reduced the adverse renal effects of DM1, consistent with the observed body weight data.

### Investigation of the mechanism by which DM1-mPEG/HER-PGNSs exposed to NIR illumination induced apoptosis in vivo

**Fig. 9:**
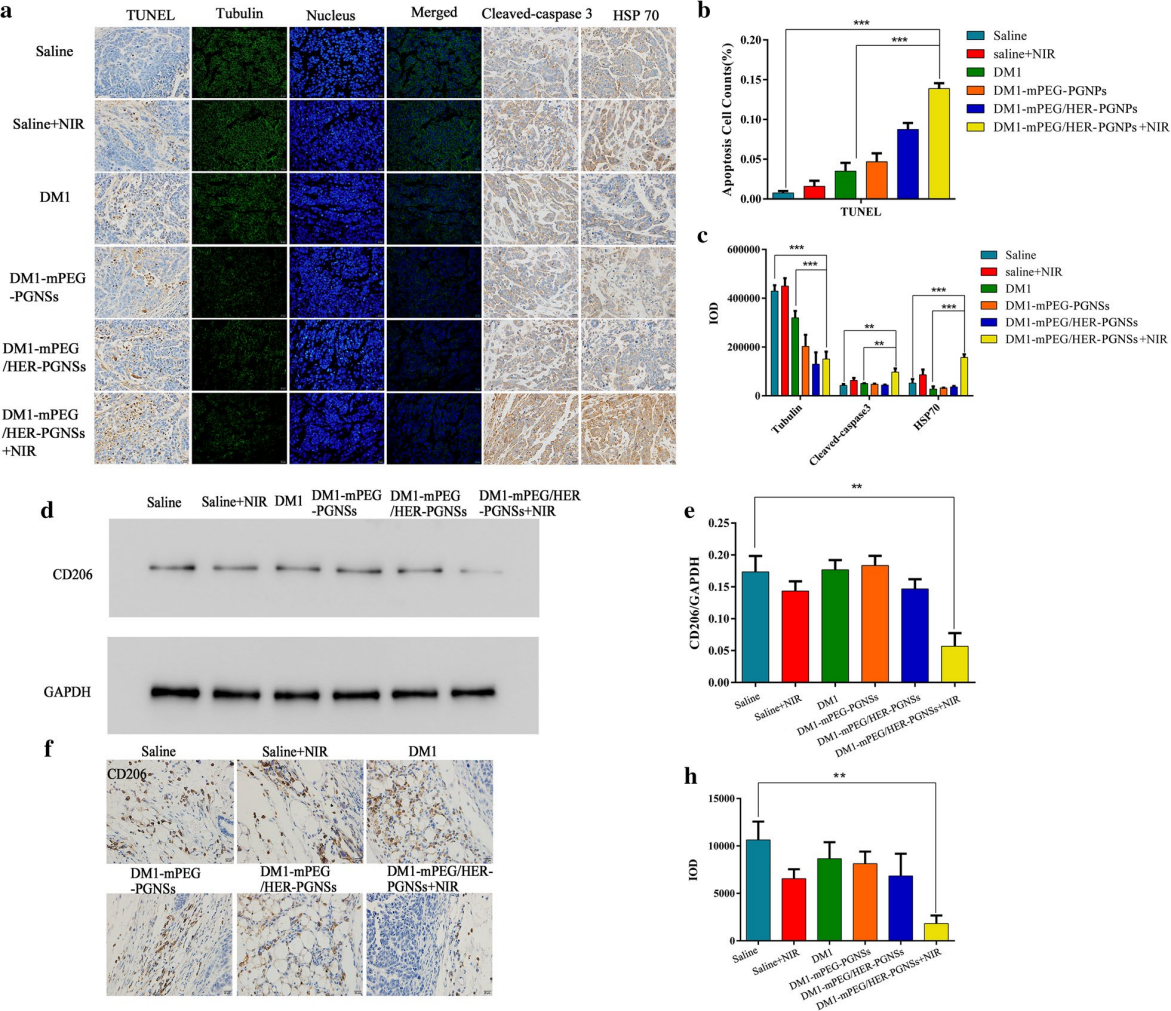
Apoptosis mechanism related protein detected by western blotting, immunofluorescence and immunohistological staining. **a** TUNEL assay of apoptosis in tumours and the immunofluorescent detection of tubulin, cleaved-caspase3 and HSP70; **b**, **c** apoptosis cell counts of TUNEL and IOD value of tubulin, cleaved-caspase 3 and HSP70; **d**, **e** Effect of NIR treatment on the inhibition of tumour-associated macrophages M2 polarization by western blotting; and **f**–**h** The immunohistological staining of CD206 in tumours. (^**^p < 0.01, ^***^p < 0.001)

To investigate the mechanism by which the PGNSs induce apoptosis, tumour tissues were subjected to a TUNEL assay and evaluations of the expression of tubulin, cleaved caspase-3 and HSP70. The TUNEL assay results revealed that treatment with DM1-mPEG/HER-PGNSs + NIR induced apoptosis at a higher and more rapid rate than other free drugs or NIR alone, which proved the beneficial effects of this combined chemo-thermotherapy. Immunofluorescent and immunohistochemical staining were used to detect apoptosis-related proteins, including tubulin, cleaved caspase-3 and HSP70. DM1 may inhibit tubulin polymerization by acting on the unique maytansine binding site on tubulin, which is located at the longitudinal tubulin–tubulin interfaces within microtubules [67]. Immunofluorescent tubulin staining (Fig. 9a, b) revealed lower expression in the tumours of drug-loaded PGNS-treated mice than in the tumours of saline-treated mice, which confirmed the role of DM1 in tumour therapy. Moreover, the levels of cleaved caspase-3 and HSP70 were evaluated to determine the mechanism of apoptosis induced by thermotherapy [68, 69]. Caspase is a classical protease that mediates cell apoptosis via disintegration, and caspase-3 is considered a key protease that induces a cascade of irreversible apoptotic reactions [70]. As shown in Fig. 9c, the expression of cleaved caspase-3 increased by 2.27- and 2.19-fold (*p* < 0.01) in the DM1-mPEG/HER-PGNSs + NIR-treated group relative to the saline- and DM1-mPEG/HER-PGNSs-treated groups, respectively, indicating that thermotherapy could activate the caspase-3-mediated apoptosis pathway [69]. However, the specific mechanism by which this pathway becomes activated will require further verification.

HSPs participate in apoptosis, thermotolerance, immuno-modulation and resistance to tumour and virus infection [71]. On one hand, exposure to heat or other stimuli induces the upregulation of HSPs to protect the cells from damage. On the other hand, HSPs stimulate the immune system via cognate pathways [72, 73] and the HSP-Ag peptide complex pathway [74] to enhance anti-tumour immunity. As shown in Fig. 9a, the levels of HSP70 increased significantly in both the DM1-mPEG/HER-PGNSs + NIR and the saline + NIR groups, consistent with the results of earlier western blotting experiments (Fig. 3i). These results suggest that NIR can upregulate the expression of HSP70, and that the SPR properties of PGNS can strengthen this effect. The higher level of HSP70 in the treatment group indicates that the increase in temperature induced by PGNS-mediated thermotherapy is sufficient to cause thermotolerance, while the balance between HSP-mediated heat resistance and anti-tumour immunity warrants further research.

Interestingly, we found that DM1-mPEG/HER-PGNSs + NIR sufficiently suppressed the pro-tumour M2 macrophage phenotype according to a western blotting assay (Fig. 9d–e) and immunohistochemical staining for CD206, a typical marker of M2 macrophages (Fig. 9f–h). This suppression was also observed in the saline + NIR group, suggesting that photothermal therapy alone could inhibit tumour-associated M2 macrophage polarization. Tumour-associated M2 macrophages secrete proangiogenic factors and immunosuppressive factors such as transforming growth factor-β (TGF-β), human leukocyte antigen-G (HLA-G) and interleukin-10 (IL-10), which promote malignancy and enhance cellular invasion [75, 76]. Therefore, the ability of photothermal therapy to inhibit M2 macrophages could be used as an adjuvant to immunotherapy that would overcome immunosuppression.

In summary, the synergistic anticancer effect (i.e., 1 + 1 > 2) of chemo-photothermal therapy has been demonstrated in this study. First, hyperthermia induced by photothermal agents in the tumour region upon specific NIR irradiation can not only kill cancer cells directly but also serve as a thermal trigger of controlled drug release and facilitate cell membrane permeability to enhance drug uptake [77]. Second, chemo-photothermal therapy can help eradicate tumours completely, and studies have demonstrated its excellent ability to overcome multi-drug resistance [78]. Hyperthermia has been reported to increase vascular permeability within tumour tissues, promoting drug enrichment and enhancing chemotherapeutic outcomes [79]. Chen et al. showed that photothermal effects could induce the expression of the heat shock protein trimer and reduce the expression of the exporter Pgp and mutant form of p53 to prevent doxorubicin efflux and increase doxorubicin sensitivity in MCF-7/ADR cells [80]. Finally, the molecular mechanisms contributing to the efficacy of chemo-photothermal therapy, including the effects on multiple signalling pathways in cancer cells, are not well explored and warrant further research.

### In vitro and in vivo analyses of anti-metastasis effects

**Fig. 10:**
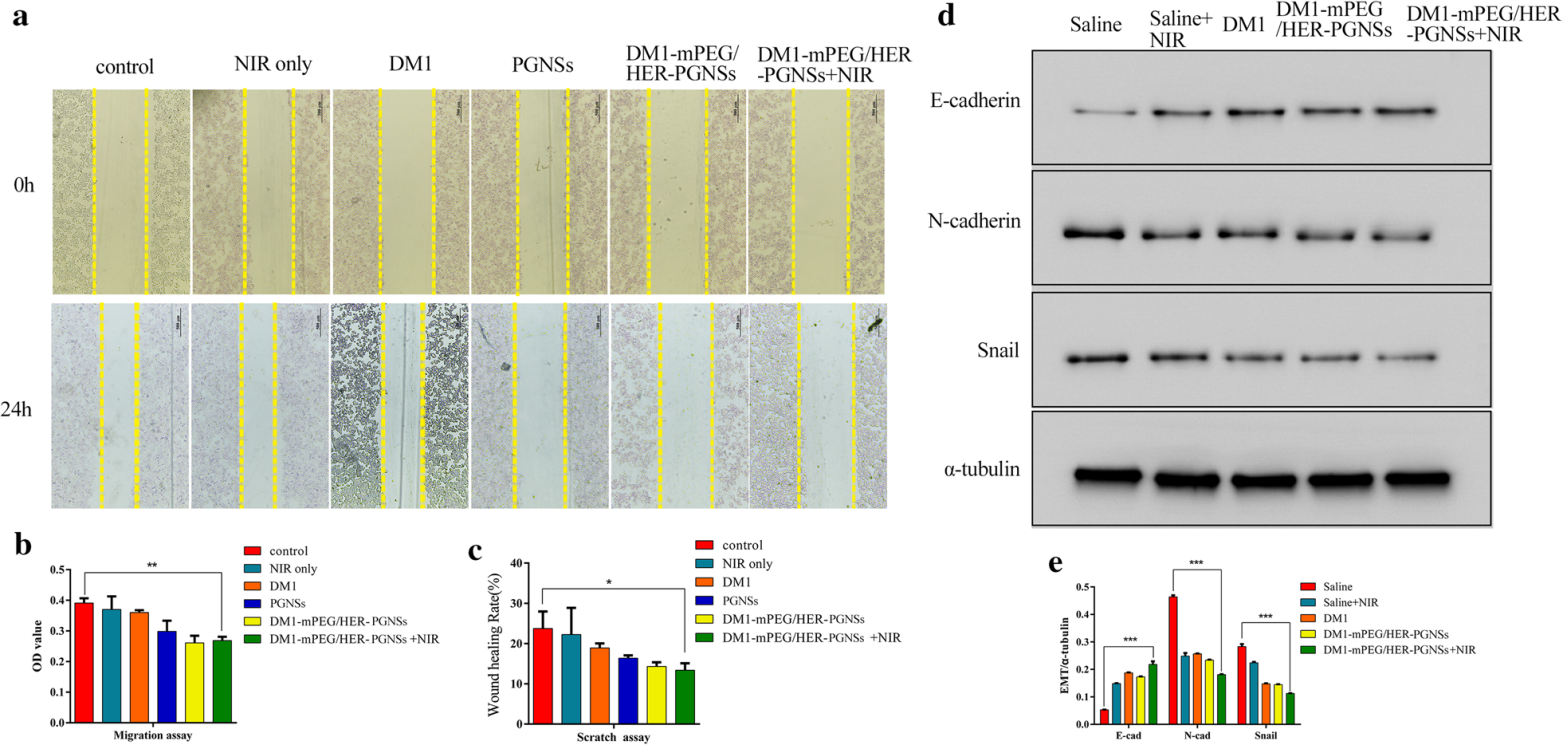
The effect of PGNS on the scratch assay and migration of SK-BR-3 cells. **a** The images of the scratch assay captured by an inverted fluorescence microscope (with 4× magnification, scale bar = 500 μm). **b** The wound healing (%) of a scratch assay calculated by Image J. **c** The OD value of cells migrated through the membrane in the migration assay; and **d**, **e** Western blotting of E-cadherin, N-cadherin and snail proteins in tumour tissues (^*^p < 0.05, ^**^p < 0.01, ^***^p < 0.001)

Scratch and migration assays were performed to determine whether the PGNS formulation or NIR treatment could inhibit cancer cell migration. Earlier studies reported that gold nanoparticles could reduce cancer cell migration by damaging the actin cytoskeleton, intercellular tight junctions and cell–cell adhesions [81]. Yang et al. reported that cells migrate more rapidly in the presence of positively charged Au NPs (including nanospheres and nanorods) and more slowly in the presence of mPEG Au NPs (neutrally charged coating) and poly(acrylic acid) Au NPs (negatively charged coating), compared with control cells cultured without Au NPs [82]. Our in vitro results (Fig. 10a–c) demonstrate that PGNSs significantly inhibited cell wound healing and migration relative to the negative control (*p* < 0.05). Next, tumours were harvested, and the expression of proteins associated with the epithelial–mesenchymal transition (EMT) was evaluated to verify the anti-metastatic effects of treatment. EMT is an essential mechanism underlying cancer metastasis and drug resistance in tumours [83]. The downregulation of E-cadherin, a cell adhesion molecule, is a critical indicator of the EMT [84]. Previous research implied that gold nanoparticles could block the EMT by enhancing the expression of epithelial markers such as E-cadherin [85, 86]. We demonstrated (Fig. 10d–e) that chemo-thermal treatment significantly enhanced E-cadherin expression and suppressed N-cadherin and Snail expression in tumour tissues exposed to the free drug, while NIR treatment alone also upregulated the expression of E-cadherin. This effect was slightly stronger in tumours exposed to targeted modified PGNSs due to the increased cellular uptake. These results indicate that our PGNS-based drug delivery system could be used as an anti-metastatic agent.

## Conclusion

In summary, we have synthesized a novel dual-targeted nanocarrier for delivering drugs to tumours, based on a porous gold nanoshell modified by mPEG and HER, to achieve a precise attack of tumours. We used DM1, a potent chemotherapeutic drug, as the model drug in our investigation of the synergistic chemo-photothermal therapeutic efficacy of these nanoparticles, as well as of their effects as contrast agents in enhanced CT/PA/PT imaging of breast cancers. Consistent with our previous findings, we observed that PEGylated PGNSs prolonged the circulation time in vivo while increasing the targeting efficacy via the EPR effect. We further confirmed that dual modification with mPEG and HER significantly enhanced the targeting of the PGNSs to Her-2-overexpressing breast cancers. Both in vitro and in vivo antitumour studies demonstrated that the DM1-mPEG/HER-PGNSs exhibited therapeutically relevant heating and significant thermo-chemotherapy capacities without inducing obvious organ damage. Simultaneously, in vivo experiments demonstrated that mPEG/HER-PGNSs yielded a stronger CT/PA contrast effect and enhanced PT imaging during photothermal therapy. To date, therapeutic nanotechnology research efforts have concentrated on drug delivery, whereas relatively little is known about the molecular mechanisms by which nanoparticles act as anti-tumour agents. In this study, our immunofluorescent, immunohistochemical and western blotting analyses revealed that chemo-photothermal therapy induced apoptosis in breast cancer cells by activating the caspase-3 and HSP70 pathways. Meanwhile, we observed the suppression of M2 macrophages in tumours, suggesting a potential immunotherapeutic use of these PGNSs. The anti-metastatic function of chemo-photothermal therapy was further demonstrated by the enhanced expression of E-cadherin both in vitro and in vivo. These results indicate that a synergistic strategy involving chemo-photothermal therapy may represent an effective approach to antitumour therapy. Furthermore, the surface-bound antibodies and targeted molecules and drugs could be changed to enable the use of our unique nanocarriers in the treatment of other cancer types.

## Methods

### Preparation of DM1-mPEG/HER-PGNSs

**Fig. 11:**
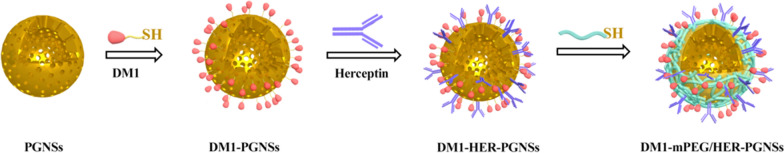
Scheme illustration of DM1-mPEG/HER-PGNSs

DM1-mPEG/HER-PGNSs was prepared as in Fig. 11. The detailed steps were in the supplementary materials. In brief, firstly, the prepared DM1-PGNSs were diluted with PBS, then herceptin was dissolved in PBS and added into the above DM1-PGNSs, when after stirring overnight, the sample solution was centrifuged for 20 min at a speed of 8000 rpm and the precipitate was redispersed with PBS. Secondly, an excess of mPEG-SH was added into the above resuspended solution and reacted for 8 h. Finally, the DM1-mPEG/HER-PGNSs were obtained in the precipitation after centrifugation for 20 min at a speed of 8000 rpm. Then, the precipitates DM1-mPEG/HER-PGNSs were collected and re-dispersed for further use.

### Characterization of mPEG-PGNSs, mPEG/HER-PGNSs, DM1-PGNSs and DM1-mPEG/HER-PGNSs

The particle size distribution of mPEG-PGNSs, mPEG/HER-PGNSs, DM1-PGNSs and DM1-mPEG/HER-PGNSs (prepared as described in the supporting information) were determined by dynamic light scattering (DLS) on a Zetasizer Nano-ZS90 device (Malvern Instruments, UK). The SPR absorption spectra ranging from 400 to 1000 nm were obtained using a UV–vis spectrophotometer (UV1200, Agilent, USA) at 25 °C. The morphologies and sizes of the PGNSs were examined using TEM (HT7700, Japan) with a CCD camera operating at an accelerating voltage of 100 kV. FTIR spectroscopy (Nicolet iS10, Thermo Fisher, USA) was used to define the formation of the Au–S bonds between mPEG and the PGNSs. The synthesized PGNSs and mPEG/HER-PGNSs were subjected to XRD analysis using a D8 Advance X-ray Diffractometer (Bruker Analytical Instruments Pvt. Ltd., Germany).

### Photothermal transformation abilities and redox sensitivity-dependent release of DM1 by DM1-mPEG-PGNSs and DM1-mPEG/HER-PGNSs

One milliliter of PGNSs, DM1-mPEG-PGNSs or DM1-mPEG/HER-PGNSs (Au concentration: 15 μg mL^−1^) was placed in a silica dish and irradiated with an 808-nm NIR laser at an intensity of 3 W cm^−2^ for 10 min. The temperature changes and images were recorded using an infrared thermal camera (FLIR Systems, Inc., USA). PBS was used as a negative control. To calculate the photothermal conversion efficiency, an aqueous solution of PGNSs was irradiated with an 808-nm laser (3 W cm^−2^) for 500 s. After turning off the laser, the temperature was measured using an infrared thermal image instrument at 20-s intervals throughout the experimental period. Furthermore, the photothermal conversion efficiency (η), an important indicator of the photothermal conversion ability, was calculated according to the equation below (Eq. 3) [87]:

3 $$ \eta = \frac{{hS\left( {T_{\max } - T_{amb} } \right) - Q_{dis} }}{{I\left( {1 - 10^{{ - A_{808} }} } \right)}} $$

where *h* is the heat transfer coefficient, *S* is the superficial area of the container, *T*_*max*_ is the maximum temperature of the solution, *T*_*amb*_ is the ambient temperature, *I* is the laser power and *A*_808_ is the absorbance of PGNSs at a wavelength of 808 nm. The sample system time constant τ_s_ was obtained from the cooling period (after 500 s) versus the negative natural logarithm of θ using Eqs. 4 and 5.

4 $$ t = - \tau_{s} ln\theta $$

5 $$ \theta = \frac{{T - T_{amb} }}{{T_{\max } - T_{amb} }} $$

Here, *hS* was determined according to the equation below (Eq. 6):

6 $$ hS = \frac{{mC_{water} }}{{\tau_{s} }} $$

where *m* is the mass of the solution and *C*_*water*_ is the heat capacity of water (4.2 J g^−1^). Moreover, *Q*_*dis*_ is the thermal diffusion value to the environment, as given by Eq. 7:

7 $$ Q_{dis} = hS(T_{{\max \left( {water} \right)}} - T_{amb} ) $$

To verify whether repeated laser irradiation would attenuate the photothermal transduction abilities of the modified and unmodified PGNSs, a 3-min irradiation course was performed nine times, and the transduction ability was analyzed as described above.

DM1 release was measured at 37 °C and a shaking speed of 100 rpm. Sample release was detected under three conditions based on PBS solutions (pH 7.4) containing 0.01% SDS: no other additives, 20 mM GSH or 20 mM GSH + NIR. Samples (0.5 mL) of media were collected at predetermined time intervals for 12 h, and the concentrations of the released DM1 were determined using high-performance liquid chromatography. Different reduction states were simulated by adding GSH. To evaluate whether NIR illumination would trigger DM1 release, the particles were subjected to 808-nm NIR irradiation at 3 W cm^−2^ for 5 min prior to sample collection.

### Stability study of PGNSs, DM1-mPEG-PGNSs and DM1-mPEG/HER-PGNSs

Using fetal bovine serum (FBS) to simulate the microenvironment, PGNSs, DM1-mPEG-PGNSs and DM1-mPEG/HER-PGNSs were incubated at 37 °C after mixing with 50% FBS, these mixtures (2 mL) were taken respectively at predetermined time intervals over 96 h and the SPR absorption spectra were obtained using a UV–vis spectrophotometer. In addition, PGNSs, DM1-mPEG-PGNSs and DM1-mPEG/HER-PGNSs were stored at 4 °C, samples (1 mL) were taken at predetermined time intervals during 28 days and irradiated using an 808 nm NIR laser at an intensity of 3 W cm^−2^ for 10 min, respectively. The temperature change was measured by a digital thermometer.

### The uptake kinetics of DM1-mPEG/HER-PGNSs

Both SK-BR-3 cells with the Her-2 receptor highly-expressed and MCF-7 cells with the Her-2 receptor lowly-expressed were used to determine the uptake kinetics of DM1-loaded nanoparticles. Cells were seeded in 6-well plates as stated previously; then the cells were incubated with PGNSs, DM1-mPEG-PGNSs and DM1-mPEG/HER-PGNSs; the medium removed after incubation for 2 h, 4 h, 12 h and 24 h; and 150 μL of RIPA was added. The cell lysates were then collected and centrifuged at 1000 rpm for 5 min. Protein and Au content were detected using the BCA Protein Assay Kit and ICP-MS, respectively, and the Au content in per unit of protein was calculated.

### Biodistribution in vivo

The animals were treated according to the ethical guidelines of China Pharmaceutical University after obtaining approval from the Animal Welfare and Research Ethics Committee of China Pharmaceutical University (No. 20190515-007). To establish the orthotopic tumour model, 1 × 10^7^ mL^−1^ SK-BR-3 cells in 100 μL of PBS were injected into the fourth left mammary fat pad (MFP) of each female BALB/c mouse. After 2 weeks, the tumour-bearing mice were intravenously administered with free SH-PEG-Cy7 or PGNSs, mPEG-PGNSs and mPEG/HER-PGNSs modified with SH-PEG-Cy7. In vivo fluorescence imaging was conducted using an IVIS Spectrum in vivo imaging system at 4 and 8 h post-injection. The tumours and main organs were dissected for ex vivo imaging. All of the isolated tissues were weighed, washed with PBS, dissolved in chloroazotic acid and digested using a microwave protocol. The Au levels in the tumours and organs were then determined by ICP-MS.

### In vivo computed tomography imaging

Enhanced CT images were acquired using a CT scanner (Iweon Multimodality system, SIEMENS) at 80 kVp, 500 μV and an integration time of 250 ms [44]. SK-BR-3 tumour-bearing female nude mice (n = 3) were scanned before and 12 h after receiving an injection of iohexol, PGNSs, mPEG-PGNSs or mPEG/HER-PGNSs (Au in all formulations: 8.5 mg mL^−1^).

### In vivo photoacoustic and photothermal imaging

SK-BR-3 tumour-bearing female nude mice (n = 3) were subjected to PA imaging using a Vevo Lazr device (VS-11946, VisualSonics, Canada). The images were acquired before and at different time points after the injection of saline, PGNSs, mPEG-PGNSs or mPEG/HER-PGNSs (Au in all formulations: 1.6 mg mL^−1^). A 735-nm excitation wavelength was used according to the results of a full-wavelength in vitro scan (Additional file 1: Figure S6). Next, the whole tumour region was irradiated with an NIR laser (808 nm, 3 W cm^−2^, 5 min) 24 h after each intravenous injection. Photothermal images were acquired using a handheld thermal camera (FLIR E5, FLIR Systems Inc.).

### In vivo antitumour efficacy

Five-week-old female BALB/c nude mice were implanted orthotopically with SK-BR-3 cells. Two weeks later, the mice were randomly divided into six groups (n = 5 per group) to receive the following treatments: saline, saline + NIR, free DM1, DM1-mPEG-PGNSs, DM1-mPEG/HER-PGNSs and DM1-mPEG/HER-PGNSs + NIR (equivalent DM1 dose: 800 μg kg^−1^). All formulations were injected intravenously via the tail vein every third day. In the indicated groups, the whole tumour region was irradiated with an NIR laser (λ = 808 nm, 3 W cm^−2^, 5 min) 24 h after each injection. The efficacy and safety of the treatments were assessed by measuring the tumour volumes and body weights, respectively. All of the mice were sacrificed after 20 days of therapy, and the organs were extracted.

### Investigation of the in vivo apoptotic mechanism

Tumour tissues were dissected from the above-described mice and subjected to a TUNEL assay, immunohistochemical and immunofluorescent detection of HSP70, cleaved caspase-3 and tubulin and western blotting of CD206.

### In vivo anti-metastasis related protein detection

Western blotting was used to detect the expression of EMT-related proteins, including E-cadherin, N-cadherin and Snail, in the tumour tissues dissected from the above-described mice.

### Statistical analysis

Statistical differences were evaluated using a Student’s *t*-test, and *p* values of < 0.05, < 0.01 and < 0.005 were considered indicative of statistically significant differences (indicated by *p < 0.05, **p < 0.01, and ***p < 0.001). All analyzed data are presented as the mean ± standard deviation. The analyses were performed using SPSS 24.0 (IBM Corp., USA).

## Supplementary Information

**Additional file 1.** Supplementary experimental section and results.

## Data Availability

The datasets used and analyzed during the current study are available from the author on reasonable request.
