# Correction to: A DM1-doped porous gold nanoshell system for NIR accelerated redox-responsive release and triple modal imaging guided photothermal synergistic chemotherapy

**DOI:** 10.1186/s12951-021-00844-1

**Published:** 2021-04-27

**Authors:** Pengcheng Xu, Ru Wang, Wenqian Yang, Yanyan Liu, Dongsheng He, Zixuan Ye, Daquan Chen, Yuan Ding, Jiasheng Tu, Yan Shen

**Affiliations:** 1 Department of Pharmaceutics, China Pharmaceutical University, Nanjing, China; 2 Chia-Tai Tianqing Pharmaceutical Group Co. Ltd., Nanjing, China; 3 School of Pharmacy, Yantai University, Yantai, 264005 China

## Correction to: *J Nanobiotechnol* (2021) 19:77 https://doi.org/10.1186/s12951-021–00824-5

Following publication of the original article [1], the authors identified inadvertent errors in Fig. 9a. Errors in the merged images of the DM1-mPEG/HER-PGNSs group and the DM1-mPEG/HER-PGNSs + NIR group, being in reverse order were found, which were possibly made during image compilation. The corrected Fig. 9 and the corrected figure caption are given below. The correction of these figures does not affect the results and conclusion. All authors agree to these corrections and apologize for these errors.

The incorrect and correct Figure 9 are published in this Correction article. The original article has been updated.

Figure 9 before correction: (Fig. 9a contained an error caused by the reverse order before submission):

**Fig. 9:**
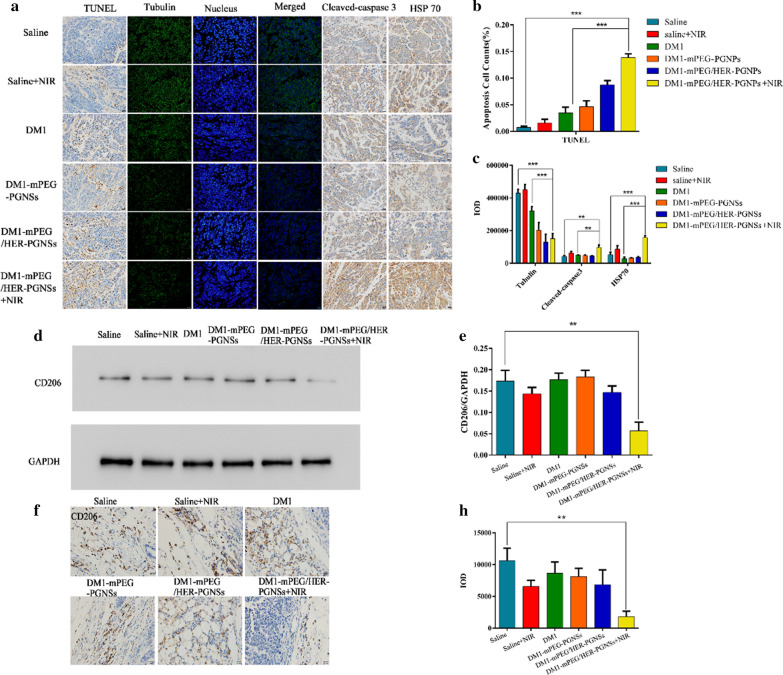
Apoptosis mechanism related protein detected by western blotting, immunofluorescence and immunohistological staining. **a** TUNEL assay of apoptosis in tumours and the immunofluorescent detection of tubulin, cleaved-caspase3 and HSP70; **b, c** apoptosis cell counts of TUNEL and IOD value of tubulin, cleaved-caspase 3 and HSP70; **d, e** Effect of NIR treatment on the inhibition of tumour-associated macrophages M2 polarization by western blotting; and **f–h** The immunohistological staining of CD206 in tumours. (**p < 0.01, ***p < 0.001)

Corrected Fig. 9:

**Fig. 9:**
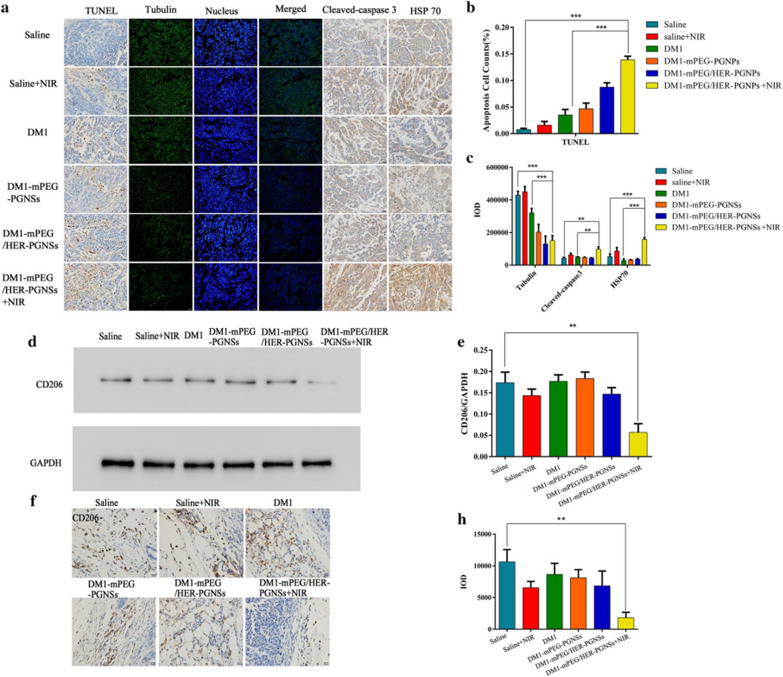
Apoptosis mechanism related protein detected by western blotting, immunofluorescence and immunohistological staining. **a** TUNEL assay of apoptosis in tumours and the immunofluorescent detection of tubulin, cleaved-caspase3 and HSP70; **b, c** apoptosis cell counts of TUNEL and IOD value of tubulin, cleaved-caspase 3 and HSP70; **d, e** Effect of NIR treatment on the inhibition of tumour-associated macrophages M2 polarization by western blotting; and **f–h** The immunohistological staining of CD206 in tumours. (**p < 0.01, ***p < 0.001)
