# Supplementary material for: A DM1-doped porous gold nanoshell system for NIR accelerated redox-responsive release and triple modal imaging guided photothermal synergistic chemotherapy
Source: J Nanobiotechnology. 2021 Mar 19;19:77. doi: 10.1186/s12951-021-00824-5 (PMC7976706; doi:10.1186/s12951-021-00824-5)
Supplement: Supplementary file 1 — Additional file 1. Supplementary experimental section and results. [file 12951_2021_824_MOESM1_ESM.docx]

**Additional Information**

**1 Experimental section**

**1.1 Preparation of PGNSs**

The preparation of PGNSs was conducted as previously described[[1](#_ENREF_1)]. Briefly, a total of 100 μL of 0.4 M aqueous cobalt chloride (CoCl_2_), 2 mL of 0.05 M aqueous sodium citrate and 100 mL of a 20% poly-N-vinylpyrrolidone (PVP K30) solution were added to 100 mL of ultrapure water in a three-necked round-bottom flask. The solution was pumped down for 10 min to extract the air and 1 mL of freshly prepared 0.1 M sodium borohydride (NaBH_4_) was added to the solution and stirred for 15 min once the solution turned gray, then pumping was stopped and a total of 300 μL of 25 mM HAuCl_4_ was injected. After that, the particles were exposed to air and the solution was shaken aggressively until the color completely changed to green. The prepared PGNSs were centrifuged for 20 min at 10000 rpm using the centrifuge (KDC-140HR, AnHui Ustc ZonKia Scientific Instruments Co., LTD). The precipitate was collected and re-dispersed for further research.

**1.2 Preparation of mPEG-PGNSs**

The preparation of mPEG-PGNSs was conducted as previously described[[1](#_ENREF_1)]. mPEG-SH was covalently grafted to the surface of the Au nanoparticles. An excess of methoxypoly(ethylene glycol)thiol (mPEG-SH, Mw = 5000) was added to the PGNSs synthesized above and the solution was stirred for 8 h to allow for the sulfhydryl group, which is known to have a strong affinity for Au, to react with PGNSs. The excess mPEG-SH was removed during centrifugation at 10,000 rpm for about 30 min, when the precipitated mPEG-PGNSs were collected and re-dispersed for further research.

**1.3 Preparation of dual modified mPEG/HER-PGNSs**

Herceptin dissolved in PBS was added into the mPEG-PGNSs previously prepared, after stirring overnight at room temperature, and the mPEG/HER-PGNSs were obtained through centrifugation for 20 min at a speed of 10,000 rpm using a centrifuge. The unconjugated Herceptin in the supernatant was detected with a BCA Protein Assay Kit (JiangSu KeyGen Biotech Company) and the connection ratio of Herceptin to Au was calculated with the details described in the supporting information.

**1.4 Preparation of DM1-PGNSs**

DM1-PGNSs were prepared through a direct-stirring reaction[[2](#_ENREF_2)]. Briefly, DM1 at a concentration of 1 mg mL^-1^was added to the PGNSs at a concentration of 100 μg mL^-1^, after stirring for 8 h at room temperature and the solution was centrifuged for 20 min at a speed of 10,000 rpm to remove free DM1 from the supernatant, then the DM1-PGNSs were obtained in the precipitate. The amount of free DM1 in the supernatant was determined by high performance liquid chromatography (HPLC) at 252 nm. DM1 loading capacity (LC) was estimated using the indirect method by determining the amount of unbound DM1 in the supernatant according to the equation 1:

$\boldsymbol{LC}\mathbf{=}\frac{\mathbf{total DM1 added-DM1 in supernatant}}{\mathbf{total Au used+total DM1 added-DM1 in supernatant}}\boldsymbol{\times100}$ **(1)**

**1.5 The detection of Au-S bond formed in mPEG-PGNSs**

Solid powders of PGNSs and mPEG-PGNSs were prepared by freeze-drying. Then, the FTIR spectra of PGNSs, mPEG-SH, and mPEG-PGNSs in the range of 900–4500 cm^−1^were recorded using an FTIR spectrometer (Nicolet iS10, Thermo Fisher, USA) in KBr pellets.

**1.6 The connection ratio between Herceptin and** **Au for mPEG/HER-PGNSs and DM1-mPEG/HER-PGNSs**

The unreacted Herceptin in the supernatant was detected using the BCA Protein Assay Kit, the standard solution and the BCA working solution were prepared according to the instruction of the BCA Protein Assay Kit. Then, a standard curve of absorbance versus the concentration of protein was obtained, and the amount of unreacted Herceptin in the supernatant was determined from the curve. The connection ratio of Herceptin and PGNSs was calculated according to the following formulation (equation 2):

$\mathbf{connection ratio}\mathbf{=}\mathbf{（}\mathbf{1}\mathbf{-}\frac{\boldsymbol{m}_{\boldsymbol{s}}}{\boldsymbol{m}_{\boldsymbol{t}}}\mathbf{）}\boldsymbol{\times100\%}$ **(2)**

where the m_s_ represents the amount of the Herceptin in the supernatant and the m_t_ represents the total amount of Herceptin added.

**1.7 Evaluation of Her-2 gene expression by real time-PCR**

MCF-7 cells, SK-BR-3 cells, BT474 cells and MDA-MB-453 cells were collected for RNA extraction by using TRIzol reagents (Invitrogen, USA) according to the instructions of the manufacturers. Briefly, 1mL of TRIzol reagent was added to the cell samples and pipetted until they were transparent. Then, 0.2 mL CHCl_3_ was added and the samples vortexed. After standing for 3 min, the samples were centrifuged for 15 min at 12000g at 4°C*.* Equal volumes of isopropanol were added to the supernatant and centrifuged for 10 min at 12000 g at 4°C. The supernatant was discarded carefully and the pellet resuspended in 1 mL of 70% ethanol. After centrifuging twice, the RNA precipitate was dried by vacuum or air for 5 min and resuspended in 30–50 µL of RNase-free water. The RNA content and purity were determined by measuring the absorbance at 260 nm and 280 nm. cDNA synthesis was performed using a First Strand cDNA Synthesis Kit (Thermo Fisher). PCR reactions were prepared using a Real time PCR Master Mix (SYBR Green, TOYOBO, Japan). The Her-2 gene expression levels (primers are listed in Table S1) in different cells were analyzed by real time PCR. All samples were normalized to GAPDH.

**Table S1.** Primer sequences of Her-2 and GAPDH.

| Gene | Forward Primer sequences(5′-3′) | Reverse Primer sequences(5′-3′) |
| --- | --- | --- |
| Her-2 | 5^’^-TTGAGTCCATGCCCAATCCC-3^’^ | 5^’^-CCTCTGCTGTCACCTCTTGG-3^’^ |
| GAPDH(Internal reference gene) | 5^’^-TGTTGCCATCAATGACCCCTT-3^’^ | 5^’^-CTCCACGACGTACTCAGCG-3_’_ |

**1.8 Examination of Her-2 protein by Western blotting**

Her-2 protein expression in the cells (MCF-7 cells, SK-BR-3 cells, BT474 cells and MDA-MB-453 cells) was examined. Cells were cultured and lysed using Lysis Buffer (KeyGen, China) to collect protein extraction. Protein concentration in the cell lysates was measured with a standard BCA method. Equal amounts of the proteins were loaded into the wells of the SDS-PAGE gel, along with a molecular weight marker. The gel was run at 90 V and the protein was transferred from the gel to the membrane. The membranes were blocked with 5% non-fat powdered milk in a Tris-buffered saline Tween (TBST) buffer. The membranes were incubated overnight at 4 °C with anti-Her-2 and anti-GAPDH and then incubated with a secondary antibody in blocking buffer containing 5% non-fat powdered milk at room temperature for 1-2 h. The membranes were washed and stained for gel imaging analysis (G:BOX ChemiXR5, SYNGENE).

**1.9 Examination of Her-2 protein by immunofluorescence detection**

The cell samples (MCF-7 cells, SK-BR-3 cells, and BT474 cells) were fixed in 4% paraformaldehyde for immunofluorescent examination. They were blocked with two drops of 3% H_2_O_2_-methanol at room temperature for 10 min. In additional, the cell slides were incubated with 50-100 μl normal goat serum for 20 min and 50 μl primary antibody for 2 h. Then, they were incubated with the FITC conjugated secondary antibody for 1h at 37℃. After staining with DAPI, fluorescent intensity was observed by a microscope.

**1.10 The cytotoxicity of PGNSs, mPEG-PGNSs and mPEG/HER-PGNSs**

A previous study [[1](#_ENREF_1)] proved that single mPEGylation doesn’t increase the toxicity of plain PGNSs, so here the MTT assay was used to investigate whether or not dual modified PGNSs by Herceptin and methoxy polyethylene glycol increased cytotoxicity. The LO2 cells were seeded in a 96-well plate at a density of 5000 cells per well. After cell adhesion, the LO2 cells were treated with increasing concentrations of PGNSs, mPEG-PGNSs and dual modified PGNSs (mPEG/HER-PGNSs), and after 24 h of incubation, MTT was added and incubated for 4 h. Then, the medium was removed and 150 μL of DMSO was added to each well and the optical density was measured at a wavelength of 570 nm using a Microplate Reader (POLARstar Omega, Germany). The relative cell viability (%) was calculated according to the equation below (equation 3)[[3](#_ENREF_3)].

$\boldsymbol{cell viability=}\frac{\boldsymbol{OD}_{\boldsymbol{sample}}\boldsymbol{-}\boldsymbol{OD}_{\boldsymbol{blank}}}{\boldsymbol{OD}_{\boldsymbol{control}}\boldsymbol{-}\boldsymbol{OD}_{\boldsymbol{blank}}}\boldsymbol{\times100\%}$ (3)

Where the OD_sample_ is the absorbance of the cells incubated with the nanoparticle sample; the OD_blank_ is the absorbance of the blank medium without cells and the OD_control_ is the absorbance of the blank medium with cells.

**1.11 Cell apoptosis assay**

The induction of apoptosis in SK-BR-3 cells and MCF-7 cells were evaluated by means of the Annexin V-FITC/PI apoptosis assay kit (Jiangsu KeyGEN BioTECH Corp., Ltd, China) using flow cytometry (BD pharmingen, bioscience). Briefly, cells were seeded in 6-well plates at a density of 5×10^5^ cells per well and were cultured at 37 °C/5% CO_2_ for 24 h. Then, the medium was replaced with free DM1, DM1-mPEG-PGNSs and DM1-mPEG/HER-PGNSs, and on top of that, one more DM1-mPEG/HER-PGNSs group was set and the cells were illuminated with 808 nm laser at 3 W cm^-2^ for 10 min after incubation for 6h. After total incubation after 24 h, the cells were trypsinized and collected, washed twice with PBS, centrifuged at 2000 rpm for 5 min and re-suspended in a 500 μL binging buffer, followed by 5 μL of an Annexin V-FITC solution and 5 μL of a PI solution. After incubation for 15 min at room temperature, cell apoptosis was analyzed by flow cytometry. Additionally, the presence of a heat shock protein 70 (HSP 70) was determined using western blot analysis. Briefly, 2.0 mL of 5× 10^5^ SK-BR-3 cells were suspended in 12-well plates and incubated for 24 h, then, cells in 12-well plates were treated with 2 mL of PGNSs, mPEG-PGNSs and mPEG/HER-PGNSs at a Au concentration of 50 μg mL^-1^ in the presence or absence of NIR laser irradiation. After being lysed, cells were analyzed by SDS-PAGE (Bio-Rad, Power Supplies Basic, USA) and transferred to a nitrocellulose filter membrane. After being blocked with 5% skimmed milk, the membrane was incubated for 12 h with a mouse monoclonal HSP 70 primary antibody (Jiangsu KeyGEN BioTECH Corp., Ltd, China) followed by a peroxidase-linked secondary antibody (Jiangsu KeyGEN BioTECH Corp., Ltd, China). The protein expression level was detected using Gel-Pro32 software (Media Cybernetics, USA).

**1.12 Competitive inhibition of Her-2 receptor**

Her-2 receptor over-expressed SK-BR-3 cells were chosen to conduct the following experiments. Briefly, SK-BR-3 cells were seeded in 6-well plates at a density of 5×10^5^ cells per well and cultured at 37 °C/5% CO_2_ for 24 h, then the medium was replaced with mPEG/HER-PGNSs. Meanwhile, excess Herceptin was added to block the Her-2 receptor before adding the mPEG/HER-PGNSs as the control group. The medium was removed after incubation for 2, 4, 12, 24 and 48 h and each well was washed with ice cold PBS three times, then the cells were lysed with 150 μL of RIPA (Beyotime, China) and collected. The supernatant was used to detect the protein content with the BCA Protein Assay Kit after centrifugation at 1000 rpm for 5 min. The remaining solution was used to detect Au content using ICP-MS (Thermo Fisher, USA) after digestion with the MDS-6G Microwave Digestion System (SINEO, China) and the Au content in per unit of protein was calculated and compared.

**1.13 The combination index calculation of DM1 and photothermal therapy mediated by PGNSs in SK-BR-3 cells**

SK-BR-3 cells were seeded in 96-well plates, then cells were incubated with free DM1, PGNSs and DM1-mPEG/HER-PGNSs, and the last two group cells were illuminated with a 808 nm laser at 3 W cm^-2^ for 10 min after incubation with the formulations for 6 h. After a total incubation of 24 h, the medium was removed and replaced with MTT. After incubation of 4 h, MTT was removed and 150 μL of DMSO was added to each well. The absorbance of each well at 570 nm was read using a Microplate Reader.

**1.14 Scratch assay**

SK-BR-3 cells were plated onto 12-well culture plates and scraped with a micropipette tip to create a mechanical scratch wound after 24 hours. The medium was discarded and washed with PBS, then these wells were divided into the control, NIR only, DM1, PGNS, DM1-mPEG/HER-PGNSs and DM1-mPEG/HER-PGNSs plus NIR groups. After 24 hours of incubation, the images of the plates were captured by an inverted fluorescence microscope (Ts2R, Nikon). The change of the scratch wound’s area was analyzed by utilizing ImageJ software (Rawak Software, Inc., Stuttgart, Germany) and wound healing (%) was calculated according to the equation below (equation 4).

$\boldsymbol{wound healing(\%)=}\frac{\boldsymbol{Area}_{\boldsymbol{0}\boldsymbol{h}}\boldsymbol{-}\boldsymbol{Area}_{\boldsymbol{24}\boldsymbol{h}}}{\boldsymbol{Area}_{\boldsymbol{0}\boldsymbol{h}}}\boldsymbol{\times100\%}$ **(4)**

**1.15 Migration assay**

A migration assay was conducted using Transwell inserts (Costar Transwell, 8.0-µm with Size 24 Cluster Platel). SK-BR-3 cells were suspended in 0.5% BSA medium and were seeded into the upper layer of the insert and incubated with different formulations to divide these wells into the control, NIR only, DM1, PGNS, DM1-mPEG/HER-PGNSs and DM1-mPEG/HER-PGNSs+NIR groups. The 10% FBS complete medium was placed into the lower chamber. Following an incubation period of 24 hours, the inserts were washed with PBS and fixed with cold methanol. The cells migrating through the membrane were stained for 20 minutes using 0.1% crystal violet, then decolored using 33% acetic acid to determine the absorption at 570 nm.

**1.16 *In vivo* toxicity assays**

The organ damage assays were performed by evaluating corresponding functional enzymes. At the end of the treatment, the blood samples of each mouse were collected by extracting the eyeball into an Eppendorf tube. Then, the blood samples were centrifuged at 3000 rpm for 5min to obtain plasma samples for measuring clinical parameters, including alanine aminotransferase (ALT), aspartate aminotransferase (AST), blood urea nitrogen (BUN), and creatinine (CREA) by the corresponding ELISA kid according to the standard protocols provided by the suppliers. Then, the main organs were sampled and applied for hematoxylin and eosin (HE) staining to assess adverse effects.

**2 Result and discussion**

**2.1 The detection of the Au-S bond formed in mPEG-PGNSs**

FTIR spectroscopy (Figure S1) showed that a weak band near 2550 cm^−1^ virtually existed in the mPEG-SH which confirmed the presence of the SH group, while the band was not observed in the spectra of mPEG-PGNSs that confirmed the S-Au interaction[[4](#_ENREF_4)]. The band near 2880 cm^-1^ which existed in the mPEG and mPEG-PGNSs corresponded to the stretching vibration of CH_2_ in the mPEG chain.


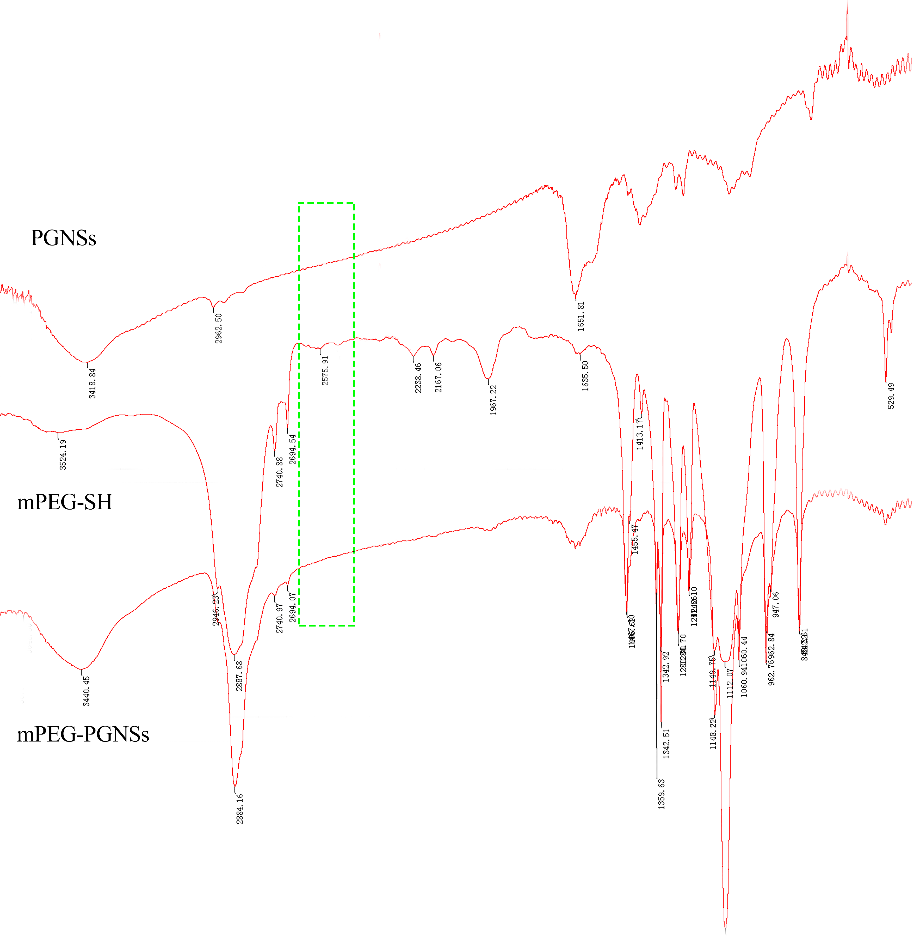


**Figure S1**. Infrared spectra of PGNSs, mPEG-SH and mPEG-PGNSs.

**2.2 The connection ratio between Herceptin and Au for mPEG/HER-PGNSs and DM1-mPEG/HER-PGNSs**

Plotting the absorption of the standard solution against the concentration of the protein, the protein standard curve was drawn in Figure S2, and we could find that good linearity between Abs and the protein concentration existed in the range of 0 to 500 μg mL^-1^ (A=0.000881*C+0.01139, R^2^=0.998). After detection of the free Herceptin in the supernatant, the connection ratio between Herceptin and PGNSs was determined and is showed in Table S2.

**Table S2.** Combined ratio of mPEG/HER-PGNSs and DM-mPEG/HER-PGNSs

|  | mPEG/HER-PGNSs | DM-mPEG/HER-PGNSs |
| --- | --- | --- |
| mHER/mAu | 38.6±6.8% | 35.1±0.3% |

**Figure S2**. The protein standard curve **2.3 Evaluation of Her-2 gene expression by real time-PCR and Her-2 protein by Western blotting and immunofluorescent detection**

Four kinds of breast cancer cells (MCF-7 cells, SK-BR-3 cells, BT474 cells and MDA-MB-453 cells) were used here to assess Her-2 gene expression with real time-PCR. As shown in Figure S3A, Her-2 protein was overexpressed in SK-BR-3 cells, BT474 cells and MDA-MB-453 cells, instead of MCF-7 cells. In addition, the gene expression in MDA-MB-453 cells was 21-fold higher than in MCF-7, while it was 4-fold and 9-fold higher for SK-BR-3 and BT474 cells, respectively.

Western blot analysis was used to test the Her-2 protein expression in order to verify the Her-2 highly-expressed cells and we found that SK-BR-3 cells, BT474 cells and MDA-MB-453 cells overexpressed Her-2 compared to MCF-7 (Figure S3B and Table S3). However, Her-2 protein expression in MDA-MB-453 cells was less than the others while for the SK-BR-3 cells it was the highest, which was different with the real time-PCR results. This may be attributed to the incomplete transcription of mRNA to Her-2 protein. As further evidence, the immunofluorescence observation (Figure S3C) was in accordance with the Western blotting results, showing the highest Her-2 expression in SK-BR-3 cells. Her-2 overlapped with the distribution of the nuclei in the SK-BR-3 cells, suggesting we could use SK-BR-3 cells as the Her-2 high-expressed cells to conduct in vitro and vivo experiments.

**Table S3**. Gray values of Her-2 protein expression among four different breast cancer cells.

|  | MCF-7 | MDA-MB-453 | BT474 | SK-BR-3 |
| --- | --- | --- | --- | --- |
| Her-2/GAPDH | 0.10±0.03 | 0.28±0.04 | 0.60±0.08 | 0.74±0.05 |

**
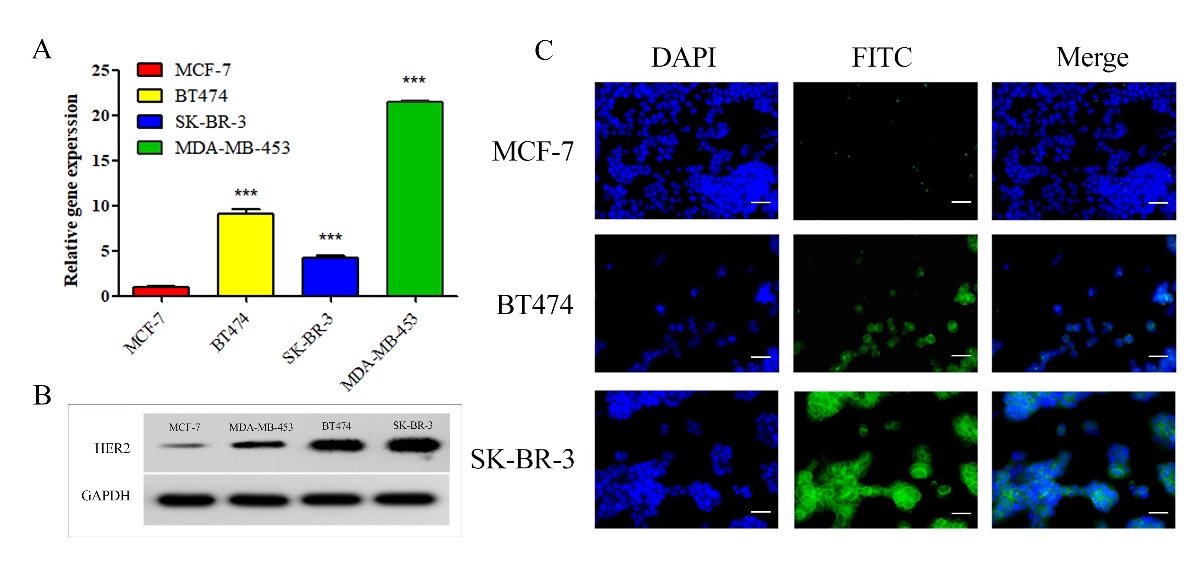
**

**Figure S3.** Evaluation of Her-2 gene and protein expression. (A) Relative gene expression of Her-2 gene by real time-PCR; (B) Western blot analysis of Her-2 protein; and (C) Immunofluorescence assay of Her-2（green fluorescence represents FITC, which indicates Her-2 receptors, blue fluorescence represents nuclei, scale bars=50 μm). *^***^p* < 0.01.

**2.3 The Stability study of mPEG-PGNSs and mPEG/HER-PGNSs**

We investigated the serum stability of mPEG-PGNSs and mPEG/HER-PGNSs, and the results are as follows (Figure S4). Both mPEG-PGNSs and mPEG/HER-PGNSs were stable within 96 hours. In addition, the structure of PGNSs is similar to that of hollow gold nanoparticles (HGNPs), and the stability of mPEG-HGNPs has been studied in our previous paper[[5](#_ENREF_5)].


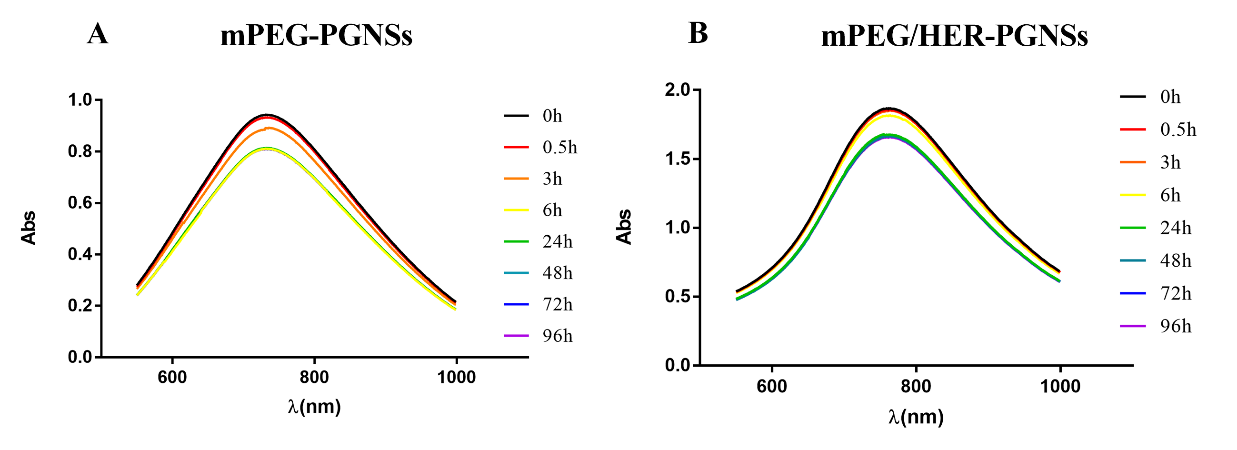


**Figure S4** The absorption spectra of mPEG-PGNSs (A), mPEG/HER-PGNSs (B) in the presence of serum

**2.4 In vitro cytotoxicity and cell apoptosis assay**

Our previous study [[1](#_ENREF_1)] proved that both PGNSs and mPEG-PGNSs had no significant influence on cell proliferation when the Au concentration was below 50 μg mL^-1^. Here, we explored the cytotoxicity of mPEG/HER-PGNSs using MTT assays with human hepatocyte cells (LO2) and the results are presented in Figure S5A. Compared with plain PGNSs, cell viability when exposed to mPEG-PGNSs and mPEG/HER-PGNSs was also higher than 90% when the Au concentration was below 50 μg mL^-1^. The cytotoxicity increased with an incremental change in Au concentration. The cytotoxicity for the mPEG-PGNSs was lower than that for the PGNSs on account of the stealth effect of PEGylation, which gives rise to the enhanced reduction of serum protein binding and reduced cellular uptake[[6](#_ENREF_6)]; this might explain the reduced cytotoxicity of the mPEG-PGNSs as less nanoparticles were internalized into the cells. However, when the concentration was above 100 μg mL^-1^, the cytotoxicity of all of the three formulations increased due to the larger concentration of Au. In conclusion, the modification of mPEG or Herceptin did not create additional toxicity to the PGNSs.

The synergistic effect of the chemotherapy and photothermal therapy in SK-BR-3 cells can be evaluated by the combination index (CI) [[7](#_ENREF_7)] using CompuSyn software (ComboSyn Inc, Paramus, NJ; [www.combosyn.com](http://www.combosyn.com/)) as the equation describes below[[8](#_ENREF_8)]:

**CI=**$\frac{\boldsymbol{(D)}_{\boldsymbol{1,50}}}{{\boldsymbol{(D}_{\boldsymbol{50}}\boldsymbol{)}}_{\boldsymbol{1}}}\boldsymbol{+}\frac{\boldsymbol{(D)}_{\boldsymbol{2,50}}}{{\boldsymbol{(D}_{\boldsymbol{50}}\boldsymbol{)}}_{\boldsymbol{2}}}$ **(5)**

Where (D_X_)_1_ and (D_X_)_2_ are the concentration of DM1 and PGNSs with NIR alone resulting in 50% growth inhibition, while (D)_1_ and (D)_2_ are the concentration of DM1 and PGNSs with NIR in the combination leading to the same growth inhibition. The values of (DX)1, (DX)2, (D)1 and (D)2 were 4.15477, 37.3929, 16.8080 and 178.738, respectively, and the combination index was 0.45164 at the IC50 value according to Figure S5B-D, which indicated the synergistic effect of DM1-mediated chemotherapy and PGNSs-mediated photothermal therapy (CI<1, =1, and >1 indicated synergism, additive effect and antagonism, respectively[[8](#_ENREF_8)]).

MCF-7 cells were used to verify the higher inhibition effect of the Herceptin modified drug-loaded PGNSs (Figure S5E). Due to the targeting effect of Herceptin modification, which promoted more DM1-mPEG/HER-PGNSs to be ingested into cells, higher cytotoxicity was observed with the lowest survival viability of 36% to SK-BR-3 cells (Figure 3G) which showed a significant difference (^*^*p*<0.05) compared to the MCF-7 cells with the lowest survival viability of 57%. Cell apoptosis is usually accompanied by a distinct set of morphological changes. As shown in the fluorescence micrographs of the nucleus in Figure S5F, the nucleus architectures were completely circular or oval with sharpened edges for the control group for both two cells. There were varying degrees of apoptosis for the different formulations of DM1 and DM1-loaded PGNSs including DM1-mPEG-PGNSs and DM1-mPEG/HER-PGNSs including the destruction and condensation of the nuclei, some even broke up into circular particles in different sizes, which appeared as bright blue hyperchromia of granular or blocky fluorescence.

The Annexin-V/FITC and PI double staining kit was used to quantify SK-BR-3 and MCF-7 cell apoptosis (Figure S5G). The total cell apoptosis rate for the DM1-mPEG/HER-PGNSs plus NIR group was ~8.4-fold and ~12.5-fold higher than the control group for MCF-7 cells and SK-BR-3 cells, respectively (Figure 3H). It was observed that the DM1-mPEG/HER-PGNSs with Herceptin modification could induce more (*p*<0.01) SK-BR-3 cell apoptosis with a higher expression of the Her-2 receptor. In addition, the combination of NIR illumination could achieve a synergistic effect in both two kinds of breast cells, which corresponded to the cytotoxic study results.


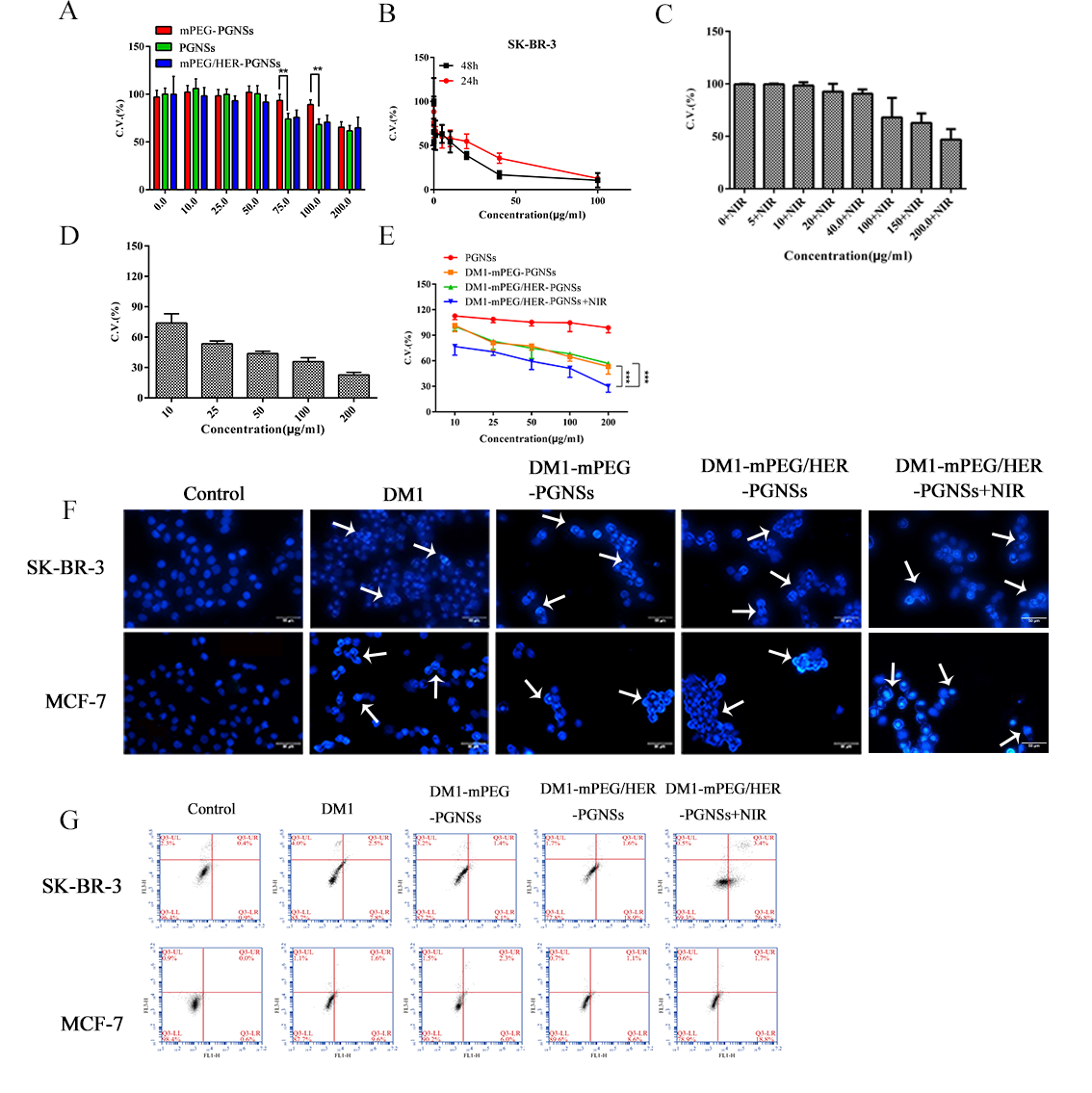


**Figure S5.** (A) LO2 cell viability when exposed to PGNSs, mPEG-PGNSs, and mPEG/HER-PGNSs for 24h; (B) SK-BR-3 cell viability when exposed to free DM1 for 24h and 48h; (C) Cell viability of PGNSs against SK-BR-3 cells; (D) Cell viability of DM1-mPEG/HER-PGNSs plus NIR against SK-BR-3 cells; (E) MCF7 cell viability when exposed to PGNSs, DM1-mPEG-PGNSs, DM1-mPEG/HER-PGNSs and DM1-mPEG/HER-PGNSs plus NIR illumination for 24h; (F) Fluorescence micrographs of SK-BR-3 and MCF7 cell nuclei labeled with DAPI (white arrows indicate morphological changes of nuclear, scale bar=50μm); and (G) Cell apoptosis evaluated with Annexin V-FITC/PI Apoptosis Detection kit. Cells were treated with medium, DM1, DM1-mPEG-PGNSs, DM1-mPEG/HER-PGNSs and DM1-mPEG/HER-PGNSs plus NIR illumination for 24 h (*^**^p<*0.01, *^***^p*<0.001*).*

**2.5 Photoacoustic phantom imaging in vitro**

Different mPEG/HER-PGNSs concentrations (0.1, 0.2, 0.4, 0.6, 0.8 and 1.0 mg mL^-1^) were imaged (Figure S6B) at a laser wavelength of 735 nm according to the result of wavelength scanning (Figure S6A). Obviously, PA intensity increased gradually with increasing concentrations of mPEG/HER-PGNSs. In addition, as shown in Figure S6C, PA intensity grew linearly with the Au concentration.


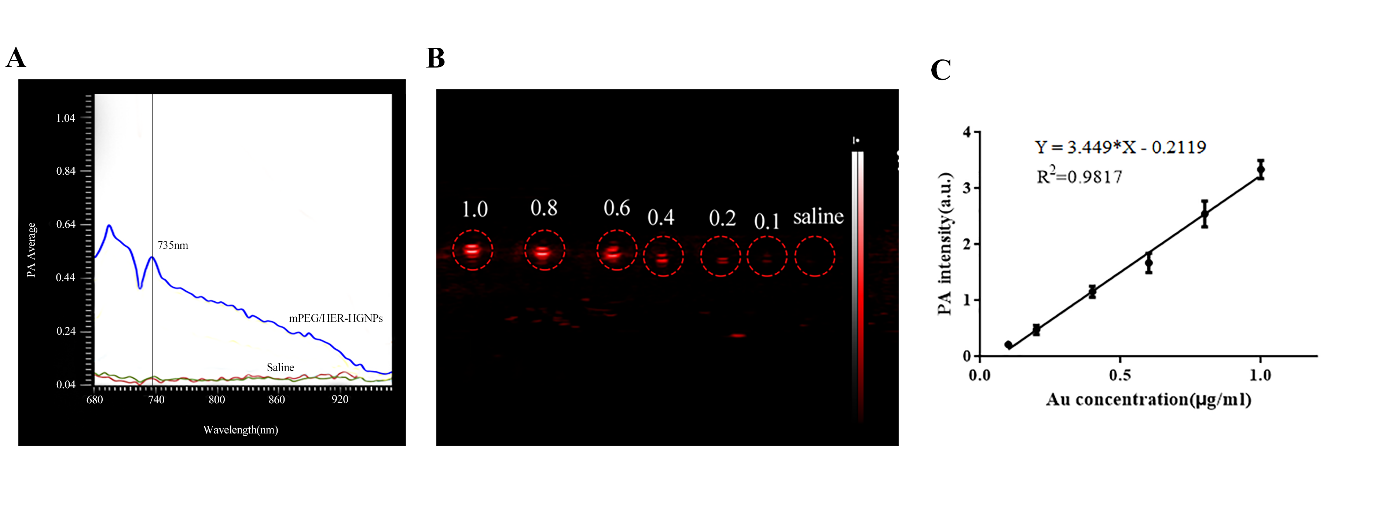


**Figure S6.** (A) PA wavelength scanning of mPEG/HER-PGNSs between 680-980nm; (B) PA images of mPEG/HER-PGNSs samples at different concentrations; and (C) Quantification of PA intensity versus mPEG/HER-PGNSs concentrations.

**2.6 In vivo toxicity assays**

The side effects of cancer chemotherapeutics on normal organs in clinical practice are an urgent problem. To evaluate the biocompatibility and toxicity of these DM1 formulations, alterations of clinical chemical parameters in mice including ALT, AST, BUN and CREA were also determined to evaluate their safety (Figure S7A). The results showed that all parameters for the different groups were substantially maintained at normal levels as well as no significant differences compared to control group were found. In addition, pathological changes in the heart, liver, spleen, lung and kidney were assayed using HE staining after treatment with different formulations. As show in Figure S7B, no obvious damage (such as inflammatory responses) in the main organs were observed during the DM1- mPEG/HER-PGNSs treatment period, while there appeared cell degeneration and necrosis in the liver tissue with the free DM1 solution. These results suggested that this delivery system did not cause remarkable damage during treatment and could decrease the side effects of DM1 to the kidney which were consistent with the weight changes because the nanoparticles could be ingested by hepatocytes then entered the small intestine through the bile duct, and were excreted by feces[[9](#_ENREF_9)].


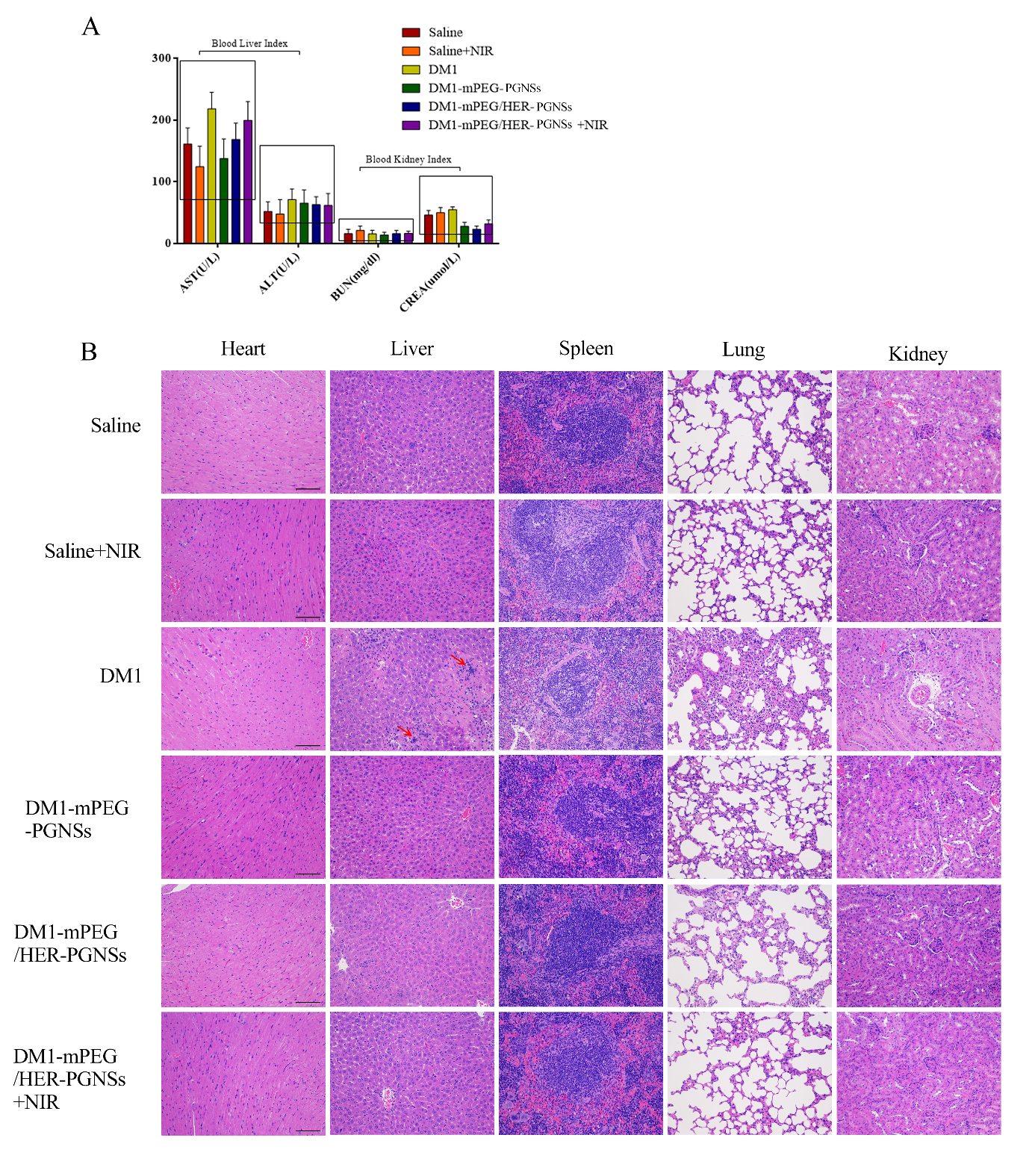


**Figure S7.** In vivo toxicity experiment. A: Liver and kidney function evaluation by detecting AST, ALT, BUN and CREA levels at the end of therapy and B: HE staining images of major organs after treatment (scale bar=100 μm, red arrow indicates damage areas).

**References**

[1] R. Wang, J. Deng, D. He, E. Yang, W. Yang, D. Shi, Y. Jiang, Z. Qiu, T.J. Webster, Y. Shen, PEGylated hollow gold nanoparticles for combined X-ray radiation and photothermal therapy in vitro and enhanced CT imaging in vivo, Nanomedicine 16 (2019) 195-205.

[2] Y. Yang, Y. Lin, D. Di, X. Zhang, D. Wang, Q. Zhao, S. Wang, Gold nanoparticle-gated mesoporous silica as redox-triggered drug delivery for chemo-photothermal synergistic therapy, J. Colloid Interface Sci. 508 (2017) 323-331.

[3] Y. Li, D. He, J. Tu, R. Wang, C. Zu, Y. Chen, W. Yang, D. Shi, T.J. Webster, Y.J.N. Shen, The comparative effect of wrapping solid gold nanoparticles and hollow gold nanoparticles with doxorubicin-loaded thermosensitive liposomes for cancer thermo-chemotherapy, 10(18) (2018) 10.1039.C7NR09083H.

[4] S. Aryal, R. B.K.C, N. Dharmaraj, N. Bhattarai, C.H. Kim, H.Y. Kim, Spectroscopic identification of SAu interaction in cysteine capped gold nanoparticles, Spectrochimica Acta Part A: Molecular and Biomolecular Spectroscopy 63(1) (2006) 160-163.

[5] R. Wang, J. Deng, D. He, E. Yang, W. Yang, D. Shi, Y. Jiang, Z. Qiu, T.J. Webster, Y. Shen, PEGylated hollow gold nanoparticles for combined X-ray radiation and photothermal therapy in vitro and enhanced CT imaging in vivo, Nanomedicine : nanotechnology, biology, and medicine 16 (2019) 195-205.

[6] J.J.F. Verhoef, T.J. Anchordoquy, Questioning the use of PEGylation for drug delivery, Drug Deliv Transl Res 3(6) (2013) 499-503.

[7] W. Lei, C. Sun, T. Jiang, Y. Gao, Y. Yang, Q. Zhao, S. Wang, Polydopamine-coated mesoporous silica nanoparticles for multi-responsive drug delivery and combined chemo-photothermal therapy, Materials Science and Engineering: C 105 (2019) 110103.

[8] Chou, T.-C., Theoretical Basis, Experimental Design, and Computerized Simulation of Synergism and Antagonism in Drug Combination Studies, Pharmacological Reviews 58(3) 621-681.

[9] H. Johnston, G. Hutchison, F. Christensen, S. Read, S. Hankin, V. Stone, A review of the in vivo and in vitro toxicity of silver and gold particulates: Particle attributes and biological mechanisms responsible for the observed toxicity, Crit. Rev. Toxicol. 40 (2010) 328-46.
